# Novel highly-multiplexed AmpliSeq targeted assay for *Plasmodium vivax* genetic surveillance use cases at multiple geographical scales

**DOI:** 10.3389/fcimb.2022.953187

**Published:** 2022-08-11

**Authors:** Johanna Helena Kattenberg, Hong Van Nguyen, Hieu Luong Nguyen, Erin Sauve, Ngoc Thi Hong Nguyen, Ana Chopo-Pizarro, Hidayat Trimarsanto, Pieter Monsieurs, Pieter Guetens, Xa Xuan Nguyen, Marjan Van Esbroeck, Sarah Auburn, Binh Thi Huong Nguyen, Anna Rosanas-Urgell

**Affiliations:** ^1^ Biomedical Sciences Department, Institute of Tropical Medicine, Antwerp, Belgium; ^2^ Department of Clinical Research, National Institute of Malariology, Parasitology and Entomology, Hanoi, Vietnam; ^3^ Department of Molecular Biology, National Institute of Malariology, Parasitology and Entomology, Hanoi, Vietnam; ^4^ Menzies School of Health Research, Charles Darwin University, Darwin, NT, Australia; ^5^ Department of Epidemiology, National Institute of Malariology, Parasitology and Entomology, Hanoi, Vietnam; ^6^ Clinical Sciences Department, Institute of Tropical Medicine, Antwerp, Belgium; ^7^ Mahidol‐Oxford Tropical Medicine Research Unit, Mahidol University, Bangkok, Thailand; ^8^ Centre for Tropical Medicine and Global Health, Nuffield Department of Medicine, University of Oxford, Oxford, United Kingdom

**Keywords:** *Plasmodium vivax*, malaria, genetic surveillance, use case, drug resistance, molecular epidemiology and population genetics, next generation sequencing (NGS)

## Abstract

Although the power of genetic surveillance tools has been acknowledged widely, there is an urgent need in malaria endemic countries for feasible and cost-effective tools to implement in national malaria control programs (NMCPs) that can generate evidence to guide malaria control and elimination strategies, especially in the case of *Plasmodium vivax*. Several genetic surveillance applications (‘use cases’) have been identified to align research, technology development, and public health efforts, requiring different types of molecular markers. Here we present a new highly-multiplexed deep sequencing assay (Pv AmpliSeq). The assay targets the 33-SNP vivaxGEN-geo panel for country-level classification, and a newly designed 42-SNP within-country barcode for analysis of parasite dynamics in Vietnam and 11 putative drug resistance genes in a highly multiplexed NGS protocol with easy workflow, applicable for many different genetic surveillance use cases. The Pv AmpliSeq assay was validated using: 1) isolates from travelers and migrants in Belgium, and 2) routine collections of the national malaria control program at sentinel sites in Vietnam. The assay targets 229 amplicons and achieved a high depth of coverage (mean 595.7 ± 481) and high accuracy (mean error-rate of 0.013 ± 0.007). *P. vivax* parasites could be characterized from dried blood spots with a minimum of 5 parasites/µL and 10% of minority-clones. The assay achieved good spatial specificity for between-country prediction of origin using the 33-SNP vivaxGEN-geo panel that targets rare alleles specific for certain countries and regions. A high resolution for within-country diversity in Vietnam was achieved using the designed 42-SNP within-country barcode that targets common alleles (median MAF 0.34, range 0.01-0.49. Many variants were detected in (putative) drug resistance genes, with different predominant haplotypes in the *pvmdr1* and *pvcrt* genes in different provinces in Vietnam. The capacity of the assay for high resolution identity-by-descent (IBD) analysis was demonstrated and identified a high rate of shared ancestry within Gia Lai Province in the Central Highlands of Vietnam, as well as between the coastal province of Binh Thuan and Lam Dong. Our approach performed well in geographically differentiating isolates at multiple spatial scales, detecting variants in putative resistance genes, and can be easily adjusted to suit the needs in other settings in a country or region. We prioritize making this tool available to researchers and NMCPs in endemic countries to increase ownership and ensure data usage for decision-making and malaria policy.

## Introduction

Despite *Plasmodium vivax* making a small contribution to the number of cases (2.9%) and deaths of the global malaria burden in 2020, outside of Africa, *P. vivax* accounts for anywhere between 18.5% of cases in the Eastern Mediterranean and 68.3% of cases in the Americas (World Health Organization, 2021). In addition, global estimates do not take latent and subpatent reservoirs into account, thus largely underestimating the impact from chronic *P. vivax* malaria on human health (Battle and Kevin Baird, 2021). Furthermore, recent studies suggest a much more widespread reservoir of *P. vivax* transmission, with a potential role for extravascular niches where the parasite can survive while it is not detected (Dechavanne et al.; Obaldia et al., 2018; Silva-Filho et al., 2020; Kho et al., 2021). There are an increasing number of reports of *P. vivax* infections now being detected with sensitive tools in Sub-Saharan Africa where it was long thought to have been absent due to the high proportion of Duffy-negative phenotype in the population (Culleton et al., 2009; Oboh et al., 2020; Quaye et al., 2021; Wilairatana et al., 2022).

Considerable progress has been made with reducing malaria cases in the last two decades, however, the rate of this reduction is stalling. Many countries moving towards malaria elimination in the next 5-10 years are making better progress with reducing *P. falciparum* cases than co-endemic *P. vivax* (Bousema and Drakeley, 2011; Douglas et al., 2013). This difference is related to key biological differences between the two parasite species. In *P. vivax*, sexual stages appear early on in the infection, even prior to clinical presentation, enabling effective transmission to mosquitoes at low-level parasitemia’s (Bousema and Drakeley, 2011; Douglas et al., 2013; Sattabongkot et al., 2004). In addition, residual *P. vivax* can be challenging to eliminate due to many subpatent and asymptomatic infections, and a hidden reservoir of hypnozoites that is difficult to target and contributes to the parasite spread across regions and borders (Moreira et al., 2015; Auburn et al., 2021; Angrisano and Robinson, 2022; Ferreira et al., 2022). In areas that are making good progress towards elimination, an increasing proportion of cases will be imported and clustered in remote and small geographical areas (Cotter et al., 2013; Auburn et al., 2021; Angrisano and Robinson, 2022). Finally, emerging antimalarial drug resistance can undermine control and elimination efforts and presents a significant risk of malaria resurgence in countries nearing elimination (Auburn et al., 2021; Ferreira et al., 2021).

The application of genetic surveillance tools is increasingly recognized for its potential to address *P. vivax* specific control and elimination challenges supporting of national malaria control program (NMCP) priorities and decision-making (Dalmat et al., 2019; Noviyanti et al., 2020). Genetic surveillance applications (use cases) relevant for *P. vivax* include drug resistant markers monitoring and predicting the spread (gene flow) of drug resistance alleles, assessing transmission intensity, identifying foci, and measuring the connectivity between parasite populations and identifying imported cases (Dalmat et al., 2019; Noviyanti et al., 2020). Most of these use cases require genetic markers with different characteristics, which can be most efficiently targeted with a single tool. While whole genome sequencing (WGS) provides the highest amount of genetic information per sample, it is not ideal for implementation in endemic countries due to the limited availability of high throughput sequencing and bioinformatic capacity at the NMCP level. In addition, blood collected for WGS requires parasite enrichment and/or removal of white blood cells to prevent high human DNA contamination. Dried blood spots (DBS) are most easily, routinely, and widely collected, but it remains challenging to obtain consistent, high-quality WGS data from low density DBS samples.

Alternatives to WGS that are more suitable for routine surveillance are amplicon sequencing tools that combine the power of Next Generation Sequencing (NGS) with a targeted approach to generate high quality sequences at high throughput. Amplicon sequencing tools apply PCR amplification to target and enrich regions of interest, with the capacity to obtain a high depth of sequencing from low density samples with human DNA contamination in a very standardized way. Ultimately, multiplex workflows can be more time and/or cost-efficient than WGS and traditional PCR-based approaches.

The most widely used genetic markers for pathogen surveillance are mutations associated with drug resistance. However, only a few markers of *P. vivax* drug resistance have been validated to-date, due to a lack of continuous *in vitro* culture and limited attention in the past from the malaria research community (World Health Organization, 2019; Buyon et al., 2021; Ferreira et al., 2021). The focus on *P. vivax* resistance markers has relied heavily on the study of orthologues of *P. falciparum* resistance associated genes, resulting in only dihydrofolate reductase (*pvdhfr)* and dihydropteroate synthase (*pvdhps)* as validated markers of resistance (Tjitra et al., 2002; Korsinczky et al., 2004; Imwong et al., 2005; Auliff et al., 2006; Marfurt et al., 2008; Buyon et al., 2021). Other markers frequently investigated include the chloroquine resistance transporter *(pvcrt)* and the multidrug resistance transporter (*pvmdr1*) (Brega et al., 2005; Suwanarusk et al., 2007). Most assays targeting these genes (or SNPs within these genes) use PCR amplification combined with Sanger sequencing or NGS (Barnadas et al., 2008; Lin et al., 2013; Tantiamornkul et al., 2018; Silva et al., 2018), although altered gene expression has also been implied in CQ resistance (Fernández-Becerra et al., 2009; Melo et al., 2014; Sá et al., 2019; Rovira-Vallbona et al., 2021).

Genetic assays that can identify the parasite genetic background to study the origin and spread of resistant lineages have offered important insights in *P. falciparum* drug resistance evolution and spread (Jacob et al., 2021), but have infrequently been used for *P. vivax* (Zeng et al., 2021). Microsatellites that are not under evolutionary pressure are popular markers for population genetic surveillance and can be used to study connectivity and transmission intensity (Anderson TJ et al., 2000; Imwong et al., 2005; Koepfli et al., 2009; Escalante et al., 2015), but they are difficult to standardize and compare between datasets (Havryliuk and Ferreira, 2009). A first *P. vivax* barcode targeting 42-SNPs in a high resolution melt assay (Baniecki et al., 2015; Ba et al., 2020; Dewasurendra et al., 2020), designed for finger-printing *P. vivax* isolates and assigning the geographic origin, was based on genomic data from only 13 isolates from 7 countries and thus had limited efficacy (Trimarsanto et al., 2019). More recently, SNP barcodes have been developed using higher numbers of genomes, which have better resolution for fine-scale population structure and combined with Identity-By-Descent (IBD) analysis, these are superior in connectivity analyses (Friedrich et al., 2016; Henden et al., 2018; Schaffner et al., 2018; Taylor et al., 2019; Fola et al., 2020). Specific SNP-barcodes for the purpose of identifying and characterizing border malaria and imported infections (Trimarsanto et al., 2019; Diez Benavente et al., 2020) have been designed, including the 33-SNP vivaxGEN-geo barcode, although so far these have been typed using WGS data and there are no targeted assays for these barcodes.

A final application of genetic tools is the characterization of recurrent infections, which in *P. vivax* is complicated by relapsing infections from activated hypnozoites that are often genetically closely related to the initial co-transmitted infections from the same mosquito bite (Bright et al., 2014) that can also be performed well with SNP barcodes and IBD-analysis (Popovici et al.; Taylor et al., 2019; Rovira-Vallbona et al., 2021).

A multiplex approach targeting many of these different markers is ideal for genomic surveillance. In this study we present a multifunctional NGS-genotyping tool (Pv AmpliSeq) for deep sequencing of targeted regions, suitable for high throughput analysis applicable to more than one use case. The Pv AmpliSeq assay combines two SNP-barcodes for between- (33-SNP vivaxGEN-geo panel) and within-country analysis, with eleven putative drug resistance genes in a highly multiplexed NGS-protocol and a quick and easy workflow. The assay was validated with *P. vivax* confirmed samples from travelers and migrants from 28 different countries spread over four continents (2012–2019) and samples collected at sentinel sites in in Central Vietnam (2015–2019). With these samples multiple use cases for genetic epidemiology with the Pv AmpliSeq assay were demonstrated.

## Materials and methods

### Samples and study settings

**Figure 1:**
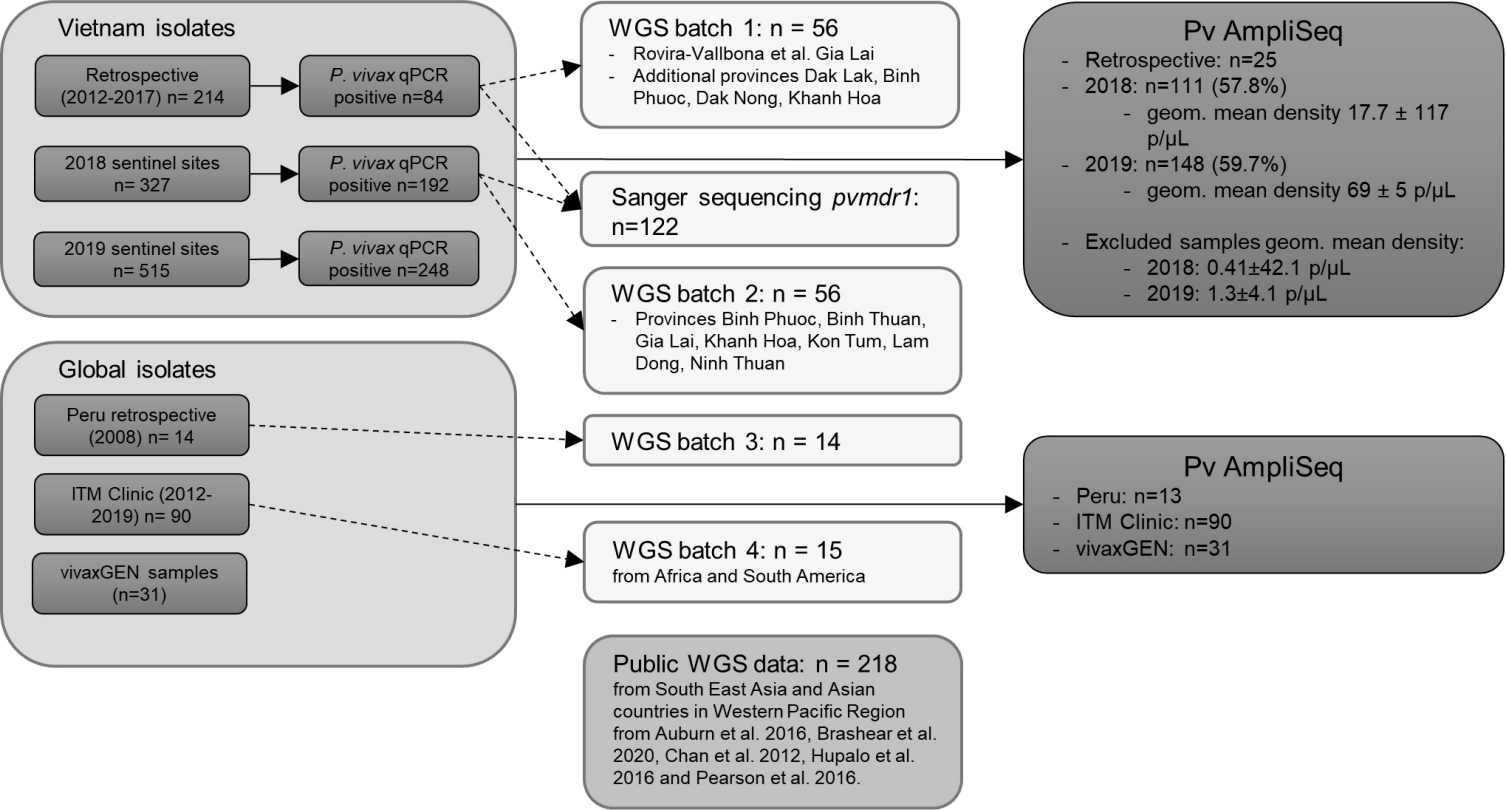
Flow-chart of sample selection for Pv AmpliSeq and WGS analysis. A database with details of samples selected for WGS can be found in **Supplementary Table 1** and for samples and controls included in the Pv AmpliSeq meta data can be downloaded from https://microreact.org/project/k86kAAWw9Z8PNeUYBj9bvh-plasmodium-vivax-ampliseq-vietnam-and-global.

Multiple sample collections were used to demonstrate different use cases with the Pv AmpliSeq assay (**Figure 1**).

### ITM clinic samples

For the use case of characterizing imported infections, blood samples (n= 90) were obtained from travelers and migrants presenting with *P. vivax* infection. These patients visited the outpatient clinic of the Institute of Tropical Medicine in Antwerp, Belgium (ITM Antwerp), or their samples were sent for diagnosis confirmation by other Belgian laboratories and hospitals to ITM Antwerp in the scope of its role as national reference laboratory. For inclusion in the Pv AmpliSeq assay we selected 90 samples from each country of origin (depending on sample volume and parasitemia by microscopy). Malaria cases were defined as the presence of Plasmodium parasites in blood smears by microscopy and confirmed by qPCR (Cnops et al., 2011). Parasitemia was quantified according to WHO standards for microscopy (World Health Organization & UNICEF/UNDP/World Bank/WHO Special Programme for Research and Training in Tropical Diseases, 2015). Fifteen of the 90 samples were analysed by WGS for comparison to the AmpliSeq data. *P. vivax* positive samples collected during a study in Peru (n=13) in 2008 (Gamboa et al., 2010) led by Universidad Peruana Cayetano Heredia (UPCH) were included to better represent South American isolates, which were poorly represented in the clinic sample set.

### Vietnam samples

For within-country analysis, *P. vivax* samples from Vietnam were included. Vietnam aims to eliminate *P. falciparum* malaria by 2025 and all human malaria by 2030 and has reduced from 4.161 confirmed cases in 2016 to 1.422 in 2020 (National Institute of Malariology, and Parasitology and Entomology, 2020a). While the predominant species causing malaria is P. falciparum (61.2%), this is followed closely by P. vivax (42.6%), which is increasing in proportion and is also causing resurgences in areas where malaria was (close to) eliminated (WHO report in National Institute of Malariology, and Parasitology and Entomology, 2020b).

Routine collections of DBS were conducted at selected sentinel site health centers (2018-2019, 6 sites/year) in the malaria-endemic provinces Binh Thuan, Binh Phuoc, Dak Nong, Gia Lai, Khanh Hoa, Kon Tum, Lam Dong, Ninh Thuan, Phu Yen, and Quang Tri to monitor uncomplicated malaria (*P. falciparum* and *P. vivax*) in Vietnam (see map in **Figure 6** for provinces with *P. vivax* cases). Individuals between 7 and 60 years old living in the area of the sentinel site clinic, visiting the clinic with suspected malaria were invited to take part in the surveillance. DBS were collected from confirmed malaria cases (positive by microscopy and/or RDT), when informed consent had been given. Parasite species was identified and quantified by qPCR [targeting varATS (Hofmann et al., 2015) or Pv mtCOX1 (Gruenberg et al., 2018)] using a standard curve of light microscopy (LM) quantified control isolates or lab-strains.

*P. vivax* infections in Vietnamese samples from 2018, were confirmed with a qPCR targeting the 18S rRNA gene with primers and a fluorescent probe labeled with HEX (**Supplementary File 2**, **Table S2**). Each reaction contained 6.25µL of TaqMan Universal Master Mix II (2x) (Applied Biosystems), 0.35µM of forward and reverse primer, 0.3µM probe, 5µL of DNA template and water to a final volume of 12.5µL. The qPCR cycling parameters for this PCR were: an initial denaturation step at 95°C for 10 min, followed by 45 cycles of 95°C for 15 seconds and 58°C for 1 minute and were run on a 7500 real-time PCR machine (Applied Biosystems).

For the Pv AmpliSeq we selected: 111/192 (57.8%) and 148/248 (59.7%) of *P. vivax* qPCR positive samples from 2018 and 2019 respectively (**Figure 1**). In provinces with few cases, all samples with parasite density ≥ 5 parasites/µL (p/µL) were selected (**Supplementary File 2**, **Table S1**), while in provinces with a high number of cases (Gia Lai 2018 & 2019, Binh Phuoc in 2018, Binh Thuan in 2019) we selected a random subset of samples stratified by commune, gender and age of the participant. Additional samples from previous collections were included from Dak Lak province (n = 6) as this province was not included in the sentinel site collections. In addition control samples (n=19) that were genotyped with other methods (WGS and/or Sanger sequencing) (Rovira-Vallbona et al., 2021).

### VivaxGEN samples

To increase the range of geographical locations, we additionally included 31 samples collected by vivaxGEN network (http://menzies.edu.au/vivaxGEN) as part of previously described clinical and cross-sectional surveys conducted between 2006-2016 (Afghanistan, Bangladesh, Bhutan, China, Colombia, Ethiopia, Malaysia, Papua New Guinea, Sudan and Thailand) and collections from returning travelers (India and Indonesia) to the Royal Darwin Hospital, Australia (Liu et al., 2014; Auburn et al., 2016; Hamedi et al., 2016; Ley et al., 2016; Wangchuk et al., 2016; Auburn et al., 2018; Hamid et al., 2018; Auburn et al., 2019; Taylor et al., 2019). In all studies, 1-5 mL venous blood samples were collected from informed, consenting patients (or a parent or guardian for individuals under 18 years of age) presenting with symptomatic, *P. vivax-*positive infection in clinical frameworks. The blood samples were subject to leukocyte depletion, followed by DNA extraction using commercial kits (Qiagen). In each country, up to three samples were selected for the Pv AmpliSeq assay (with volume ≥5 µL genomic DNA), with preference for infections with corresponding genomic data with high coverage of the *P. vivax* genome (**Figure 1**).

### Ethical considerations

The sample collections in Vietnam were approved by local ethical review boards at NIMPE and by agreement of the Ministry of Health in Vietnam. Individuals were included in this study only if they willingly signed informed consent that included an opt-in future-use clause. Presumed consent to systematically store samples for future research is established at ITM Antwerp as part of active clinical surveillance of tropical diseases. Secondary use of all samples was approved through the Institutional Review Board of the Institute of Tropical Medicine Antwerp (reference 1417/20).

Ethical approval for the vivaxGEN samples was provided by the Human Research Ethics Committee of NT Department of Health and Families and Menzies School of health Research, Darwin, Australia (HREC-09/83, HREC-2010-1431, HREC-2012-1815, HREC-13-1942, HREC-2014-2228, HREC 2012-1871), the Research Review Committee of the Institute for Medical Research and the Medical Research Ethics Committee (MREC), Ministry of Health Malaysia (NMRR-10-754-6684, NMRR-12-511-12579), the Mahidol University Faculty of Medical Technology, Thailand (MUTM 2011-043-03), the Institutional Review Board of Jiangsu Institute of Parasitic Diseases, Wuxi, China (IRB00004221), Addis Ababa University College of Natural Sciences, Ethiopia (RERC/002/05/2013), Armauer Hansen Research Institute, Addis Ababa, Ethiopia (AHRI-ALERT P011/10), the National Research Ethics Review Committee of Ethiopia (Ref.no. 3.10/580/06), International Centre for Diarrhoeal Disease Research, Bangladesh (PR-14053), the Research Ethics Board of Health, Ministry of Health, Bhutan (REBH 2012/031), the Ethics Committee of the Infectious and Tropical Disease Research Center, Hormozgan University of Medical Sciences, Iran (HUMS 9014) and the Comite Instiucional de Etica de Investigaciones en Humanos, Cali, Colombia (08–2015).

### Sample pools

Samples were mixed at equal ratios to create a pool of 3 to 5 similar samples from the same year (2018 or 2019) and province (Gia Lai, Lam Dong, Binh Thuan or Binh Phuoc). These sample pools were used as input for the library preparation to investigate feasibility of pooling samples to reduce per-sample costs by 3 to 5-fold. Allele frequencies (AF) based on pools were compared to AF based on individual sample library preparations. The library preparation for one of the 3-sample pools failed, and this pool was excluded from analysis. Within-library AF was calculated based on allele depth and averaged over the 3 or 5 combined samples in the pool. Differences between paired AF of pools and AF based on individuals samples was tested using Wilcoxon signed rank test in R.

### Controls

A high density whole blood sample collected at the ITM clinic and quantified by LM was used as a control to prepare a dilution series from 20.000 p/µL to 0.2 p/µL. In addition, a dilution series (51.6 p/µL - 0.0052 p/µL quantified by mtCOX1 qPCR) was made from the DBS-extracted DNA from a Vietnam isolate (2019 Binh Phuoc province). Both dilution series were included in the Pv AmpliSeq with and without prior selective whole genome amplification (sWGA) using 2 rounds of PCR as described previously (Cowell et al., 2017). For three replicates of the LM-quantified sWGA dilutions, the sWGA product was diluted (1:4-1:7) in order not to surpass the input amount of 100 ng of DNA.

To mimic an infection with complexity of infection (COI) of two clones we prepared mock mixtures with two single clone infection samples previously genotyped with 14 microsatellite markers (Hong et al., 2016).

As negative controls we included: 1) one uninfected human control from Belgium, 2) two qPCR *P. falciparum* & *P. vivax* negative control isolates from Vietnam, 3) one Pf3D7 laboratory strain (100 p/µL), 4) two *P. falciparum* qPCR positive, but *P. vivax* negative samples from Vietnam (2018 and 2019) and 5) one *P. knowlesi* laboratory strain () at 0.43% parasitemia.

### DNA extractions

Whole blood and DBS samples were DNA extracted using QIAmp DNA Blood Mini Kit (Qiagen, Germany) following the manufacturer’s protocol and using 1 punch (2019 samples Vietnam) or 3 punches (all other DBS samples) from DBS or an equivalent of 200 µL whole blood. Extracted DNA was resuspended in 200µl elution buffer from the kit.

### Whole genome sequencing

Samples selected for WGS analysis (n= 141) were processed in different batches (**Figure 1**). From the 56 genomes in batch 1, 28 were previously reported (Rovira-Vallbona et al., 2021) and the remaining 28 samples were retrospective sentinel site samples from Binh Phuoc, Dak Lak, Dak Nong and Khanh Hoa provinces. All samples were enriched for parasite DNA after DNA extraction using two rounds of sWGA (Cowell et al., 2017; Rovira-Vallbona et al., 2021). AMPureXP purified DNA was sent to BGI (Hong Kong) for batch 1-3 or GenomeScan (The Netherlands) for batch 4 for low-input library preparation and 150-bp paired-end sequencing on a HiSeq X Ten (Illumina) or NovaSeq instrument (Illumina) as previously described (Rovira-Vallbona et al., 2021).

FASTQ files were processed for samples from batch 1 as previously described (Rovira-Vallbona et al., 2021). Briefly, FASTQ files were trimmed using Trimmomatic to remove adapters and low-quality reads and aligned to the reference genome PvP01 using bwa mem and variants were called using HaplotypeCaller in GVCF mode followed by Joint-Call Cohort (GATK 4.1.2.0; Broad Institute) and filtered to include only biallelic SNPs in the core genome (MQ>50, QUAL>30, and combined DP ≥100). The resulting high quality SNPs (n =191849) were used in a pipeline to select SNPs for the within-country barcode where LD-pruning was conducted in 5-6 iterations by scanning over the genome in 500 bp windows to remove uninformative SNPs with pairwise LD>0.2 using the python package scikit-allel. Remaining SNPs where then filtered for a minor allele frequency (MAF) above 10%, and selectively neutral SNPs were selected by calculating Tajima’s D in 500bp windows and retaining those where |Tajima’s D|>0.5 resulting in a list of 1152 SNPs. Subsequently, the contributions of the SNPs to geospatial genetic clusters were determined using discriminant analysis of principal components (DAPC) (Jombart et al., 2010) with province populations and K-means inferred populations (n = 27) using adegenet package in R.

For *in silico* validation of the SNP barcode additional genomes from batch 2-4 were combined with the genomes from batch 1 supplemented with online available *P. vivax* genomes (n=218) (Auburn et al., 2016; Chan et al., 2012; Hupalo et al., 2016; Pearson et al., 2016) (See Supplementary file 1 for table with details and accession numbers for all genomic data incl. the genomes generated in this study). FASTQ files were aligned to the GRCh38 human reference genome using the Burrows-Wheeler aligner (bwa mem) version 0.7.17 (Li and Durbin, 2009) to remove all human reads. Unmapped reads (non-human) were subsequently aligned to the PvP01 reference genome version 46 from PlasmoDB (Auburn et al., 2016) using bwa. Variant calling was performed using HaplotypeCaller (GATK 4.1.9.0) and resulting GVCF files were merged using CombineGVCFs followed by joint-genotyping with GenotypeGVCFs. Variants were hard filtered using parameters according to GATK best practices and annotated using SnpEff (Cingolani et al., 2012). Variants with more than 50% missing genotypes in all samples were removed from analysis. Mean depth was calculated using VCFtools (Danecek et al., 2011). Samples were included in the analyses when ≥75% of barcode positions (Vietnam + vivaxGEN-geo) were genotyped (Supplementary file 1).

### Sanger sequencing *pvmdr1*

To compare Pv AmpliSeq performance to Sanger sequencing we applied a nested PCR amplification approach targeting the *pvmdr1* gene as previously described (Barnadas et al., 2008; Tantiamornkul et al., 2018). Nested PCR products were quantified by Nanodrop ND-1000 (Isogen) and sent for Sanger sequencing at a commercial facility (Genewiz, Germany). DNA sequences were aligned and compared to the *pvmdr1* reference sequences (PVP01_1010900 and PVX_080100 from PlasmoDB https://plasmodb.org/ version 43 ) using multiple sequence alignment with ClustalW in MEGA7: Molecular Evolutionary Genetics Analysis version 7.0 (Kumar et al., 2016) and contiguous sequences were made using Bioedit v.7.2.5.

### AmpliSeq library prep and bioinformatics

Primers targeting the desired regions were designed as AmpliSeq Custom Panel using the DesignStudio Software using the PvP01 reference genome by the Illumina Concierge team (Illumina, San Diego, USA). This design process makes individual amplicon testing before multiplexing unnecessary. Pv AmpliSeq library preparation was performed using AmpliSeq Library PLUS for Illumina kit (Illumina), AmpliSeq Custom Panel design (*i.e.* the primer pools as per the design) for *P. vivax* (Supplementary file 3) and AmpliSeq CD Indexes (Illumina) as per the manufacturer’s instructions. Library preparation was performed on 7 µL of DNA, undiluted in the case when DNA was extracted from DBS, or diluted within the range of 50-150 ng DNA input in the case of whole blood samples. Target regions were amplified with adjusted cycling conditions (99°CC for 2 min, followed by 21 cycles of 99°CC for 15 sec and 60°CC for 8 min) in two reactions, and subsequently combined for final library preparation according to the guidelines. Libraries were quantified using Qubit v3 High sensitivity DNA kit (Invitrogen), and subsequently diluted to 2nM with low Tris-EDTA buffer, and pooled. Denatured library pool (7 pM) was loaded on a MiSeq system (Illumina) for 2x300 paired-end sequencing (Miseq Reagent Kit v3, Illumina) with 1% PhiX spike-in (Illumina).

FASTQ files were processed with an in-house analysis pipeline on a Unix operating system computer. Reads from the demultiplexed FASTQ files were trimmed using Trimmomatic (settings: ILLUMINACLIP: 2:30:10 LEADING:3 TRAILING:3 SLIDINGWINDOW:4:15 MINLEN:36) to remove adapter sequences and poor-quality reads. Trimmed reads were aligned to the PvP01 reference genome version 46 from PlasmoDB using Burrows-Wheeler aligner (v0.7.17) with bwa mem (Li and Durbin, 2009). Alignment statistics were generated using Picard’s CollectAlignmentSummaryMetrics. Variants were called using HaplotypeCaller (GATK, v4.1.2) and individual sample- and control gVCF-files were combined to jointly call genotypes using GenotypeGVCFs. Variants were hard filtered (QUAL>30, overall DP>5000, MQ>50, QD>1.0, SOR<4, GT depth >5, as decided by browsing the distributions of these variables) and variants were annotated with SnpEff (v4.3T) (Cingolani et al., 2012), resulting in 4363 high quality genotypes (incl. all variant types, e.g. SNPs and indels). Per locus filtered depth of coverage (format field DP) was used to calculate median depth of all loci per sample or per amplicon. Aligned coverage was calculated as the number of bases-passed filter divided by the number of bases (57011 bp) targeted in the Pv AmpliSeq assay.

### Sample size and final sample inclusion criteria

For the final population level analysis of use cases we selected 394/431 (91.4%) samples with good quality data (<50% missing genotype calls, mean coverage >15) and retaining only one library of replicates (with lowest missingness; replicates included: 15 Vietnam and 4 Peru). With a sample size of 96 samples a MAF of 0.01 with 0.02 (2%) precision could be detected, and with 381 samples MAF=0.01 with (1%) precision (https://epitools.ausvet.com.au/oneproportion).

### Analysis

Allele frequencies at barcode loci were calculated from allele depths to reflect true population AF in complex infections. First, AF was calculated per locus and sample using the allele depths in R. Then, we summed the AF at each locus (SUM-AF) from all samples and then divided the SUM-AFs by the sum of within-sample AFs for all alleles at that locus.

Within-host infection complexity was assessed using within-sample F statistic (F_WS_) using the R package moimix (Lee and Bahlo, 2016), and F_WS_ ≥ 0.95 was considered a proxy for a monoclonal infection.

Population genetic analyses were performed using the 42-SNP Vietnam barcode, excluding samples missing >18 SNPs (25%). Genetic differentiation was measured as F_ST_ (Weir and Cockerham, 1984) using 1000 bootstraps with the R package DiveRsity (Keenan et al., 2013). Genetic diversity was expressed as expected heterozygosity (*He*) that was calculated [adegenet package in R (Jombart and Bateman, 2008; Jombart et al., 2011)] using diploid barcode genotypes. DAPC (Bright et al., 2014) was performed with cross-validation using the adegenet package in R, and associated allele loadings for the first four components were determined.

A likelihood-based classifier was used to predict the origin of *P. vivax* isolates using the 42-SNP Vietnam and 33-SNP vivaxGEN-geo barcodes combined and the 33-SNP vivaxGEN-geo barcode alone, using a previously reported framework (https://geo.vivaxgen.org/) and reference dataset (Trimarsanto et al., 2019). Two of the 42-SNP loci (PvP01_06_v1:45794 and PvP01_14_v1:3004298) were not included in the predictions owing to indels in the reference dataset. The comparative predictive performance of the remaining 40-SNP Vietnam and 33-SNP vivaxGEN-geo barcodes was evaluated using a stratified 10-fold cross-validation with 500 repeats, reporting the Matthews Correlation Coefficient (MCC).

A list of variants of interest (Supplementary file 4) was created including variants in drug resistance associated genes that have been reported to have a potential association with resistance through literature research (**Table 1**), and supplemented with any non-synonymous variants detected in the target genes in more than one study sample using the Pv AmpliSeq assay. Haplotypes were created by combining genotypes of major variants of interest (including previously reported genotypes and those identified contributing to the DAPC in haplotypes). Frequency tables of haplotypes and variants with confidence intervals were created using the freqtables R-package.

**Table 1:** List of potential drug resistance genes targeted in the Pv Ampliseq assay.

Chromosome	Gene name	Gene ID	Drug resistance	references
PvP01_01_v1	*pvcrt* (chloroquine resistance transporter)	PVP01_0109300/PVX_087980	CQ (putative)	(Barnadas et al., 2008; Melo et al., 2014; Joy et al., 2018; Silva et al., 2018; Tantiamornkul et al., 2018; Sá et al., 2019; Rovira-Vallbona et al., 2021)
PvP01_02_v1	*pvmrp1* (multidrug resistance-associated protein 1)	PVP01_0203000/PVX_097025	CQ, PIP (both putative)	(Bright et al., 2013; Gonçalves et al., 2014; Flannery et al., 2015; Pearson et al., 2016)
PvP01_03_v1	*pvdmt2* (drug/metabolite transporter 2)	PVP01_0312700/PVX_000585	CQ (putative)	(Flannery et al., 2015)
PvP01_05_v1	*pvdhfr* (dihydrofolate reductase)	PVP01_0526600/PVX_089950	PYR	(Tjitra et al., 2002; Hastings et al., 2005; Auliff et al., 2006; Rungsihirunrat et al., 2008; Bright et al., 2013; Flannery et al., 2015; Cubides et al., 2018; Tantiamornkul et al., 2018; Auburn et al., 2019)
PvP01_10_v1	*pvmdr1* (multidrug resistance transporter)	PVP01_1010900/PVX_080100	CQ (putative)	(Suwanarusk et al., 2007; Imwong et al., 2008; Marfurt et al., 2008; Bright et al., 2013; Melo et al., 2014; Hupalo et al., 2016; Auburn et al., 2019; Ngassa Mbenda et al., 2020)
PvP01_10_v1	*pvp13k* (phophatidylinositol 3 kinase)	PVP01_1018600/PVX_080480	ART (putative)	(Hupalo et al., 2016; Mbenda et al., 2018)
PvP01_11_v1	*pvabc-e1* (ABC transporter E family member 1)	PVP01_1103800/PVX_115370	In IBD region recurrent infections after CQ	(Rovira-Vallbona et al., 2021)
PvP01_12_v1	*pvk13* (pvkelch13)	PVP01_1211100/PVX_083080	ART (putative)	(Flannery et al., 2015; Mint Deida et al., 2018; Tantiamornkul et al., 2018)
PvP01_12_v1	*pvmdr2* (multidrug resistance protein 2)	PVP01_1259100/PVX_118100	CQ (putative)	(Cowell et al., 2018; Noisang et al., 2019)
PvP01_14_v1	*pvdhps* (dihydropteroate synthase)	PVP01_1429500/PVX_123230	SULF; CQ (putative)	(Korsinczky et al., 2004; Imwong et al., 2005; Mint Lekweiry et al., 2012; Ganguly et al., 2014; Cubides et al., 2018; Joy et al., 2018; Tantiamornkul et al., 2018; Auburn et al., 2019; Noisang et al., 2019)
PvP01_14_v1	*pvmrp2* (multidrug resistance-associated protein 2)	PVP01_1447300/PVX_124085	CQ (putative)	(Flannery et al., 2015; Cowell et al., 2017)

The assay targets the full length genes listed above with several amplicons per gene. CQ, chloroquine; PIP, piperaquine; PYR, pyrimethamine; ART, artemisinins; SULF, sulfadoxine.

PED and MAP file formats were created using VCFtools, and IBD-sharing between pairs of samples was calculated using the isoRelate package in R (Henden et al., 2018). Genetic distance was calculated using an estimated mean map unit size from *Plasmodium chabaudi* of 13.7 kb/centimorgan (cM) (Martinelli et al., 2005; Rovira-Vallbona et al., 2021). We set the thresholds of IBD at the minimum number of SNPs (n = 10) and length of IBD segments (1000 bp) reported to reduce false-positive calls using an error of 0.001. Networks of IBD sharing between individuals were created using the igraph package in R and gene flow between provinces was estimated by summing pairwise IBD by province using a custom R-script and plotted in a Sankey Diagram using the networkD3 R-package.

## Results

### Pv AmpliSeq design

As previously published general SNP barcodes (Baniecki et al., 2015) lack the resolution for small-scale population genetic and gene flow analysis within Vietnam, we designed a 42-SNP Vietnam barcode with in-country resolution using *P. vivax* genomes (n=32 from batch 1 with isolates from different provinces in Vietnam. Neutral unlinked SNPs were filtered from the genomic dataset, while we selected SNPs with a large contribution to geographical spread and genetic in the DAPC analysis. Finally, 3 SNPs per chromosome with high allele loadings in the DAPC were selected with spread over the chromosome and not too close proximity (>500 bp) to drug resistant amplicons to avoid linkage. The allele frequencies of the 42-SNP Vietnam barcode loci were checked *in silico* with a larger set of genomes from Vietnam (n=103), and neighboring countries (n=202), including online deposited genomes. In the Vietnam genomes, the majority of SNPs had a MAF>10%, with only five alleles observed at MAF<5% including one loci where we only observed the reference allele (Supplementary file 5). With minor allele frequencies varying between provinces, this 42-SNP Vietnam barcode has sufficient resolution to detect small-scale population genetic differences within the country (Supplementary file 5). In the genome dataset including samples Vietnam as well as neighboring countries in South East Asia (n=305), allele frequencies are very similar to Vietnam alone, suggesting the barcode might be applicable to a larger region (Supplementary file 5).

**Figure 2:**
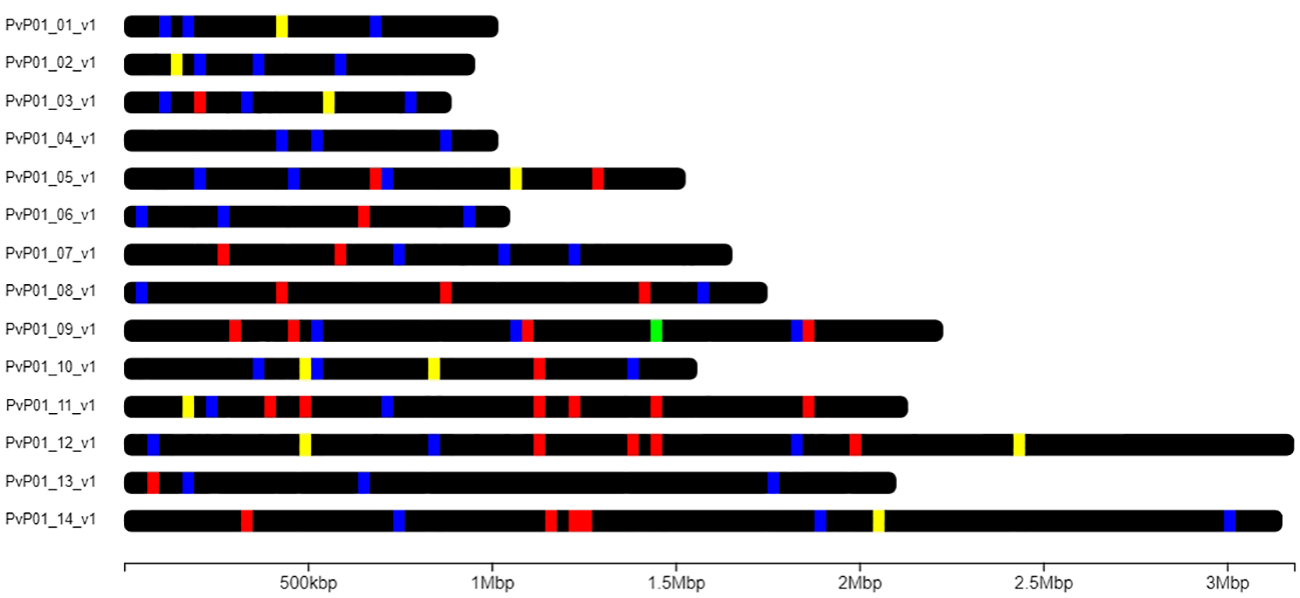
Chromosomal position of amplicons in the Pv AmpliSeq design for Vietnam. Amplicons are depicted on the 14 nuclear chromosomes of the PvP01 reference genome. Amplicons targeting drug resistance associated genes are colored in yellow, amplicons targeting the 42-SNP Barcode position in blue, the 33-SNP global barcode in red, and ama1 in green.

A second barcode, the 33-SNP vivaxGEN-geo panel, was added to the Pv AmpliSeq assay to support identification and prediction of origin of imported cases (Trimarsanto et al., 2019). This barcode targets alleles that do not vary between provinces in Vietnam and is therefore not suitable for within country analysis (Supplementary file 5). Lastly, a highly diverse region of the apical membrane antigen 1 (*pvama1*) was targeted to allow investigation of complex infections and antigenic diversity. The final assay primers, designed by the Illumina concierge team, included all desired regions (100% covered in the design) in 229 amplicons spread across the PvP01 genome (**Figure 2**) in 2 primer pools (of 115 and 114 amplicons respectively), with amplicon length varying from 161-335 bp (Supplementary file 3).

### Pv AmpliSeq assay validation

The assay was validated with 394 samples (95.6%, out of 412 successfully sequenced). The parasites in the samples originated from 28 countries, with good representation from 5 WHO regions, collected between 2008-2020 (**Table 2**). The majority of samples (66%) originated from Vietnam and 233/261 (89.3%) were routinely collected in 2018-2019 in 8 Provinces (**Table 3**). The majority of Vietnam samples were from symptomatic male patients, matching with the known local risk factors for malaria infection (overall 77.8% male of cases recorded by the NMCP in 2018 and 2019) and sample collection from suspected cases presenting to the local health facilities.

**Table 2:** Overview of good quality (coverage >15 and ≤50% genotypes missing) samples included in the Pv AmpliSeq analysis.

**WHO Region**	**Country**	**n**	**percent of total**	**years**
	unknown	2	0.51	2020 (1)
AFR (n=29; 7.4%)	Burundi	1	0.25	2019
	DRC	1	0.25	2019
	Eritrea	12	3.05	2014, 2015, 2016
	Ethiopia	13	3.30	2012, 2013, 2014, 2015, 2016, 2019
	Mauritania	1	0.25	2013
	Senegal	1	0.25	2012
AMR (n=24; 6.1%)	Brazil	2	0.51	2016, 2019
	Colombia	4	1.02	2014, 2015, 2019
	Guyana	3	0.76	2013, 2014, 2015
	Panama	2	0.51	2013
	Peru	13	3.30	2008
EMR (n=37; 9.4%)	Afghanistan	16	4.06	2012, 2013, 2015, 2016, 2017, 2018, 2019
	Pakistan	13	3.30	2014, 2015, 2016, 2017,2 018, 2019
	Somalia	1	0.25	2016
	Sudan	7	1.78	2015, 2019
EUR (n=1; 0.3%)	Spain	1	0.25	2016
SEAR (n= 27; 6.9%)	Bangladesh	3	0.76	2014, 2015
	Bhutan	2	0.51	2014
	India	15	3.81	2011, 2012, 2013, 2014, 2015, 2017, 2018
	Indonesia	5	1.27	2010, 2012, 2013, 2016, 2017
	Thailand	2	0.51	2006, 2007
WPRO (n=274; 69.5%; without VTN n= 13; 3.3%)	Cambodia	1	0.25	2018
	China	2	0.51	2009, 2011
	Malaysia	3	0.76	2014
	Papua New Guinea	5	1.27	2010, 2012, 2013, 2014, 2019
	Philippines	1	0.25	2015
	Singapore	1	0.25	2015
	Vietnam	261	66.24	2015, 2016, 2017, 2018, 2019
ALL	28 countries	394	100%	2006 - 2020

**Table 3:** Vietnam sample characteristics and epidemiological data from 2018 and 2019 (from NIMPE annual malaria report).

Province	N per province	Year	N per year	% of N total	% of N province	Recorded Pv cases NMCP	% of annual cases genetically analyzed	Pv incidence rate per 1000 pers. yr at risk	% male	median age	% with fever
Binh Phuoc	44	2016	5	1.9	11.4					20	
		2018	28	10.7	63.6	460	6.1%	0.56	89.3%	21	100%
		2019	11	4.2	25.0	209	5.3%	0.28	100.0%	25	91%
Binh Thuan	28	2018	5	1.9	17.9	73	6.8%	0.11	80.0%	17	80%
		2019	23	8.8	82.1	118	19.5%	0.21	95.7%	26	91%
Dak Lak	6	2015	6	2.3	100.0						
Dak Nong	12	2016	1	0.4	8.3						
		2019	11	4.2	91.7	60	18.3%	0.11	90.9%	30	82%
Gia Lai	102	2016	10	3.8	9.8				90.0%	19	
		2018	47	18.0	46.1	336	14.0%	0.35	93.6%	24	85%
		2019	45	17.2	44.1	448	10.0%	0.44	84.4%	26	89%
Khanh Hoa	21	2017	6	2.3	28.6						
		2018	4	1.5	19.0	34	11.8%	0.18	75.0%	32	100%
		2019	11	4.2	52.4	61	18.0%	0.54	81.8%	24	91%
Kon Tum	3	2018	3	1.1	100.0				100.0%	33	100%
Lam Dong	37	2018	15	5.7	40.5	144	10.4%	0.22	93.3%	22	80%
		2019	22	8.4	59.5	102	21.6%	0.47	95.5%	25.5	86%
Quang Tri	8	2019	8	3.1	100.0	59	13.6%	0.45	62.5%	22.5	100%
ALL			261								

The Pv AmpliSeq assay generated a high number of reads (mean 72435.9 ± 47018.86 paired reads per sample after trimming low quality reads) with a median of 98.9% [range 26.9-99.9%] of trimmed reads aligning to the PvP01 genome. Targeted regions were covered with a mean 595.7 ± 480.5 paired reads, with balanced coverage over all amplicons, with the exception of the amplicon targeting SNP PvP01_13_v1_162821 in the Vietnam barcode, the amplicon targeting the SNP SEC27 in the vivaxGEN-geo barcode and one *pvcrt* amplicon (targeting the end of the 6^th^ exon and into next intron), which had poor depth in most samples (**Supplementary File 2**, **Figures S1, S2**).

### Parasite density limits

The density limit to successfully genotype DBS samples with the Pv AmpliSeq assay was determined using two dilution series, one with an LM-quantified sample, and the other with a low density Vietnam sample quantified by mtCOX1 qPCR. Good coverage (>15) was achieved until 20 p/µL in the LM-quantified sample with the majority of genotypes called (**Supplementary File 2**, **Table S3**). In the low density sample sufficient depth of coverage was achieved up to 0.5 p/µL, but at this concentration many genotypes were missing. Therefore, with a density of >5 p/µL quantified by mtCOX1 qPCR a majority of DBS samples (92%) passed our inclusion criteria (**Supplementary File 2**, **Figure S3**). For samples with lower density, sWGA will increase coverage at the expenses of increasing genotype missingness and error rate. In this case, it is important to dilute the sWGA product prior to Pv AmpliSeq in order not to surpass the optimal input requirements from the kit.

### Minority clone detection

Two previously MS-genotyped samples were mixed at different ratios to mimic a mixed-clone infection as multiple clone infections are common in *P. vivax*. The minority clone could be detected at most variant positions (≥93.1%) when consisting of ≥10% of the mixture (**Table 4**). Minority clones at 5% and 2% could still be detected at approximately 75% of loci that were discordant between the two strains, although some minor genotypes will be missed.

**Table 4:** Detection of an artificially created mixed clone infection of two previously genotyped samples from the same period and area in Vietnam.

Ratio of mixed isolates	Mean depth	% of heterozygotes detected	
		n detected of total variant loci	% variant loci detected
50:50	212.0	56/58	96.6%
80:20	192.5	56/58	96.6%
90:10	182.2	54/58	93.1%
95:5	197.3	44/58	75.9%
98:2	174.4	46/58	79.3%

The minority clone could be detected down to 2%, although at ≥5% some genotypes of the minority clone could no longer be detected.

### Genotyping accuracy

Sequencing accuracy was high with a mean error rate of 0.013 ± 0.007 in pair-wise comparisons of replicates (n=13) with mean 0.48% ± 0.14% allelic differences at all loci detected in the assay.

When the Pv Ampliseq assay is compared to Sanger sequencing at 3 *mdr1* loci in 122 samples from Vietnam (2018), the same genotype is detected in 97.8% samples. In 2.2% of samples the AmpliSeq assay detected a mixed genotype (**Supplementary File 2**, **Table S4**), while for the Sanger sequencing we had recorded only the consensus sequence for each sample.

Compared to WGS of 58 samples, a mean 5.0% ± 2.4% discordant loci (of SNPs and indels at all AmpliSeq loci) were detected (**Supplementary File 6**). While including only homozygote genotypes (since the coverage in WGS is much lower), the mean discordance was 3.7% ± 1.8%.

### Uninfected and non-vivax plasmodium controls

Primer specificity was tested with three uninfected human blood samples (negative controls), and only detected very few reads predominantly outside the assay target regions (only 0.6% of genotypes in target region were called). This resulted only in genotypes that were below the filtering and inclusion thresholds.

As *P. vivax* co-infections with *P. falciparum* are frequent in many areas, we tested *P. falciparum* infected samples and a 3D7 laboratory strain. Similar to the uninfected controls, coverage was low (mean 9.9) for 5/5 *P. falciparum* controls with mean 99% of genotypes missing. Therefore *P. falciparum* co-infections are not expected to lead to biased results in the Pv AmpliSeq.

In addition, while human *P. knowlesi* infections are infrequent, they do occasionally occur in Vietnam, therefore, we tested a laboratory strain. Due to *P. knowlesi* and *P. vivax* genome similarities, many regions of the *P. knowlesi* genome were amplified at high coverage (303X). However, few of the *P. knowlesi* amplified regions aligned to the PvP01 reference, resulting in 86.8% missing genotypes. Therefore, this sample would not pass the inclusion criteria and would be excluded from the analysis.

### Sample pooling

We explored the option of pooling samples before the library preparation, thereby reducing the cost per sample. Allele frequencies in pools of three samples (n=5) from the same province and year in Vietnam were not significantly different from allele frequencies calculated from the same individual samples (Wilcoxon signed rank test p>0.05). However, in pools of five samples (n=2) the resulting pool population AF differs from individual sample population AF (Wilcoxon signed rank test p>0.002). Therefore, pooling of more than 3 samples is not recommended.

### Barcode performance

Both alleles were detected at all of the 42-SNPs in the Vietnam within-country barcode in the study samples. The median MAF of all barcode alleles was 0.34 [range 0.01-0.49] (Supplementary file 5). One locus was not typed as a SNP, but as indel, as there was a third allele where the two adjacent nucleotides were deleted, but this was seen at very low AF (0.005) in Vietnam. For 28.0% (230/820) of pairwise comparisons between barcode loci, significant linkage disequilibrium was observed, mostly due to linkage of alleles within provinces as a result of population substructure.

**Figure 3:**
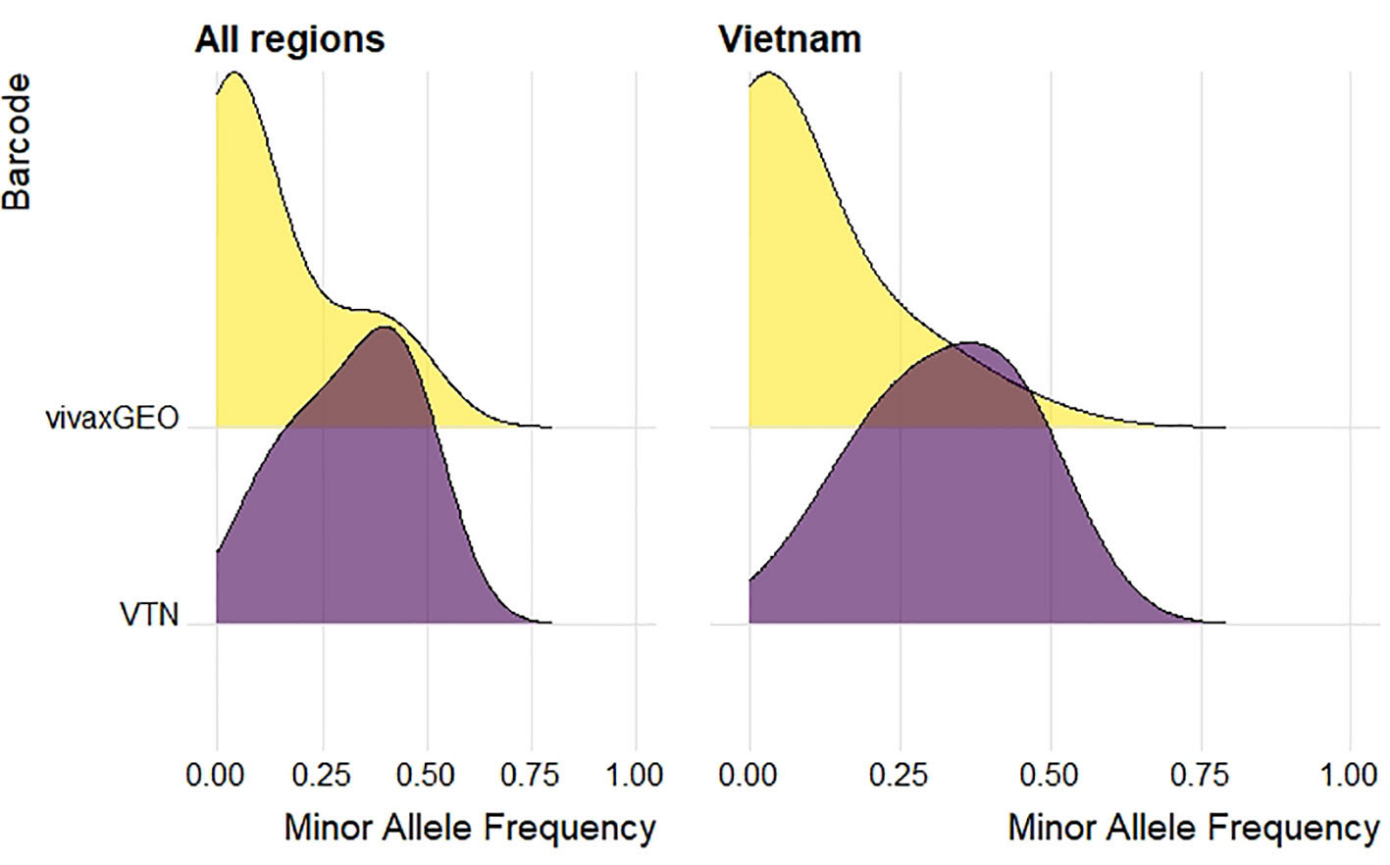
Density plot of minor allele frequencies of 33-SNP vivaxGEN-geo barcode (yellow) and 42-SNP within-country Vietnam barcode (purple) in samples tested with the Pv AmpliSeq assay. Minor allele frequencies were calculated in study samples in all regions (n=394, left) or in Vietnam only (n=261, right).

From the 33-SNP vivaxGEN-geo barcode, all loci were successfully genotyped by the Pv AmpliSeq assay. In all study samples, median minor allele frequencies of vivaxGEN-geo barcode alleles were much lower than the Vietnam barcode with median 0.04 [range 0.000-0.496] (**Figure 3**). With the lower MAF in the vivaxGEN-geo barcode it has better spatial specificity for between-country origin prediction, while the Vietnam barcode was designed to have common alleles for within-country spatio-temporal surveillance. The set of samples from travelers and migrants was selected in order to have a wide geographic representation to validate the detection of both alleles at the vivaxGEN-geo barcode loci with the Pv AmpliSeq assay. However, at two loci (PvP01_08_v1_1420994 & PvP01_11_v1_384043) we only detected the reference allele in the study samples. In PlasmoDB, the alternate alleles at these two positions were present in isolates from the Americas region (Brazil, Colombia, Mexico).

### Global *P. vivax* population description

**Figure 4:**
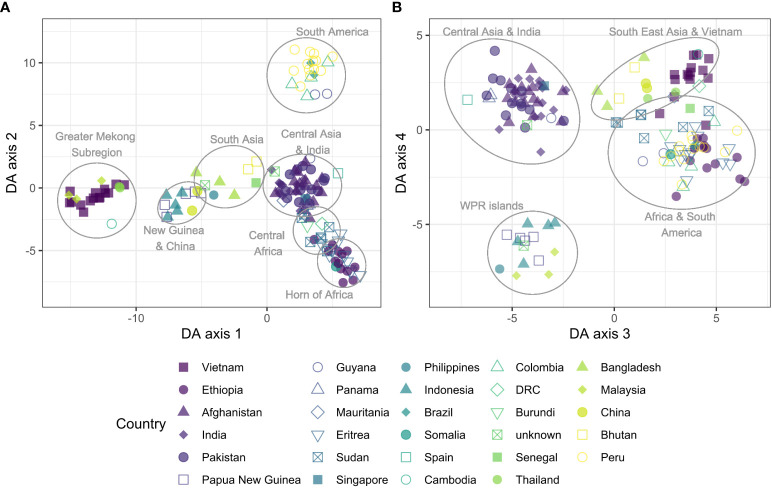
Discriminant analysis of principal components (DAPC) of global isolates (n = 148), incl. 15 samples randomly selected from Vietnam. Scatter plot of discriminant analysis (DA) eigenvalues 1 and 2 **(A)** using all biallelic SNPs detected by the Pv AmpliSeq showed a differentiation from east to west from the Pacific into Asia along the x-axis and a separation between Africa and the Americas along the y-axis. Scatter plot of DA eigenvalues 3 and 4 **(B)** groups African and American samples together, close to South East Asian and Vietnam isolates and separately cluster the WPR islands and Central Asia and India. SNPs contributing most to the DAPC are listed in **Supplementary Table 2**, **Table S5**. DAPC was performed with 20 principal components and 20 discriminants as determined through cross-validation.

Population structure of global isolates of *P. vivax* was explored using DAPC. We observed a regional separation that roughly divides the parasites in four continental groups along the first two axes (**Figure 4**), and separately clusters the Western Pacific island regions and Central Asian countries (incl. India) along the 3^rd^ and 4^th^ axes. Among the highest contributing alleles to the DAPC along the first four axes, 8/20 were from vivaxGEN-geo amplicons (**Supplementary File 2**, **Table S5**), indicating these SNPs are important for the observed geographic separation at a global level for which they were designed. Other alleles contributing to the global separation include variants in *pvmdr2*, *pvdmt2*, *pvmdr1*, *pvdhps* and *pvdhfr*.

### Use case prediction of origin of imported infections

**Figure 5:**
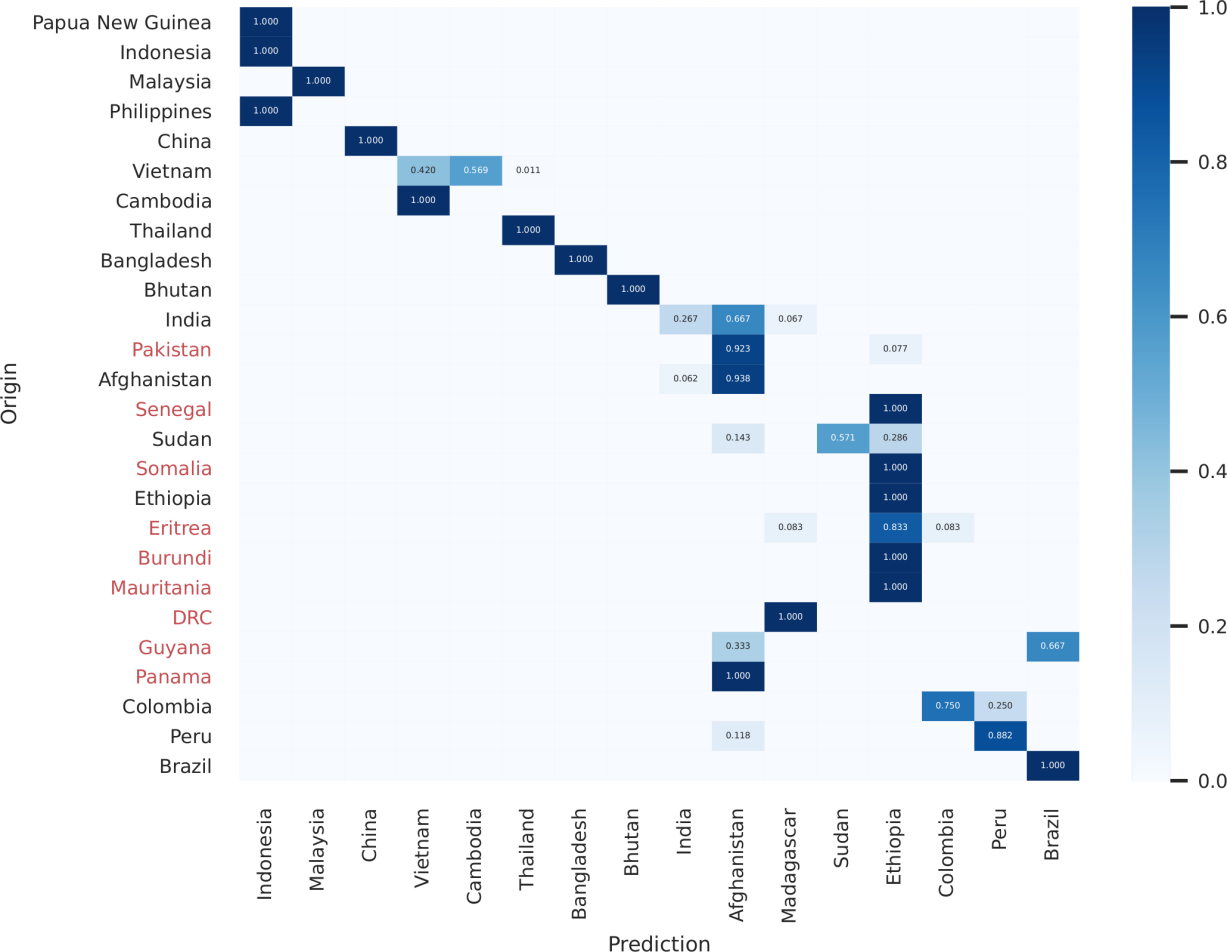
Heatmap of predicted origin in the likelihood model vs. expected origin of samples based on collection site and travel history using the 72-SNP barcode. Predicted origin of the samples was calculated with the vivaxGEN-geo likelihood classifier (Trimarsanto et al., 2019). Countries of origin that were not represented in the reference dataset and hence could not be directly predicted are indicated in red on the origin axis; these countries can be included in future iterations of the classifier to improve its accuracy. Only samples from patients who presented in malaria-endemic countries are illustrated (i.e., excluding samples from Spain, Singapore and Unknown origin samples that were present in the larger dataset). The strong diagonal trend illustrates the regional accuracy of the predictor, with most infections mapping directly to the country of origin or to neighboring countries e.g., with Vietnamese samples mapping to either Vietnam or Cambodia. In border regions with extensive parasite gene flow, many infections may not be classifiable by national (political) boundaries, and regional boundaries may prove more useful administrative units for classification.

The origin of the samples was predicted with the likelihood classifier and compared to the expected origin based on travel history or sample collection site using the 40-SNPs from the Vietnam barcode (40/42 SNPs mapped to the likelihood classifier reference dataset) and the 32-SNP vivaxGEN-geo barcode. Amongst 402 independent samples, four derived from non-endemic (Singapore and Spain) or unknown locations, and 34 derived from countries that were not represented in the reference set of the predictor tool (DRC, Eritrea, Guyana, Mauritania, Pakistan, Panama, Senegal and Somalia). Amongst the remaining 368 samples, when using the combined 72-SNP barcode, 327 samples (88.8%) were correctly predicted with either the first (186, 50.5%) or second prediction (141, 38.3%), demonstrating a high accuracy in regional spatial prediction (**Figure 5**). Notably, of the 141 samples that were mapped to the correct country of origin with the second prediction, 133 samples (94%) were samples from Vietnam or Cambodia mapping to one another as the first prediction. This pattern is also reflected with the Matthews Correlation Coefficient (MCC) scores derived from the reference genomic dataset, which are moderately low in Vietnam and Cambodia (**Supplementary File 2**, **Figure S4**). The 40-SNP Vietnam barcode, designed for population genetic analysis within country, improved the prediction compared to the vivaxGEN-geo barcode for samples originating from Vietnam and other Asian countries, but slightly reduced the prediction in South America (**Supplementary File 2**, **Figure S4**).

### Use case drug resistance globally

**Figure 6:**
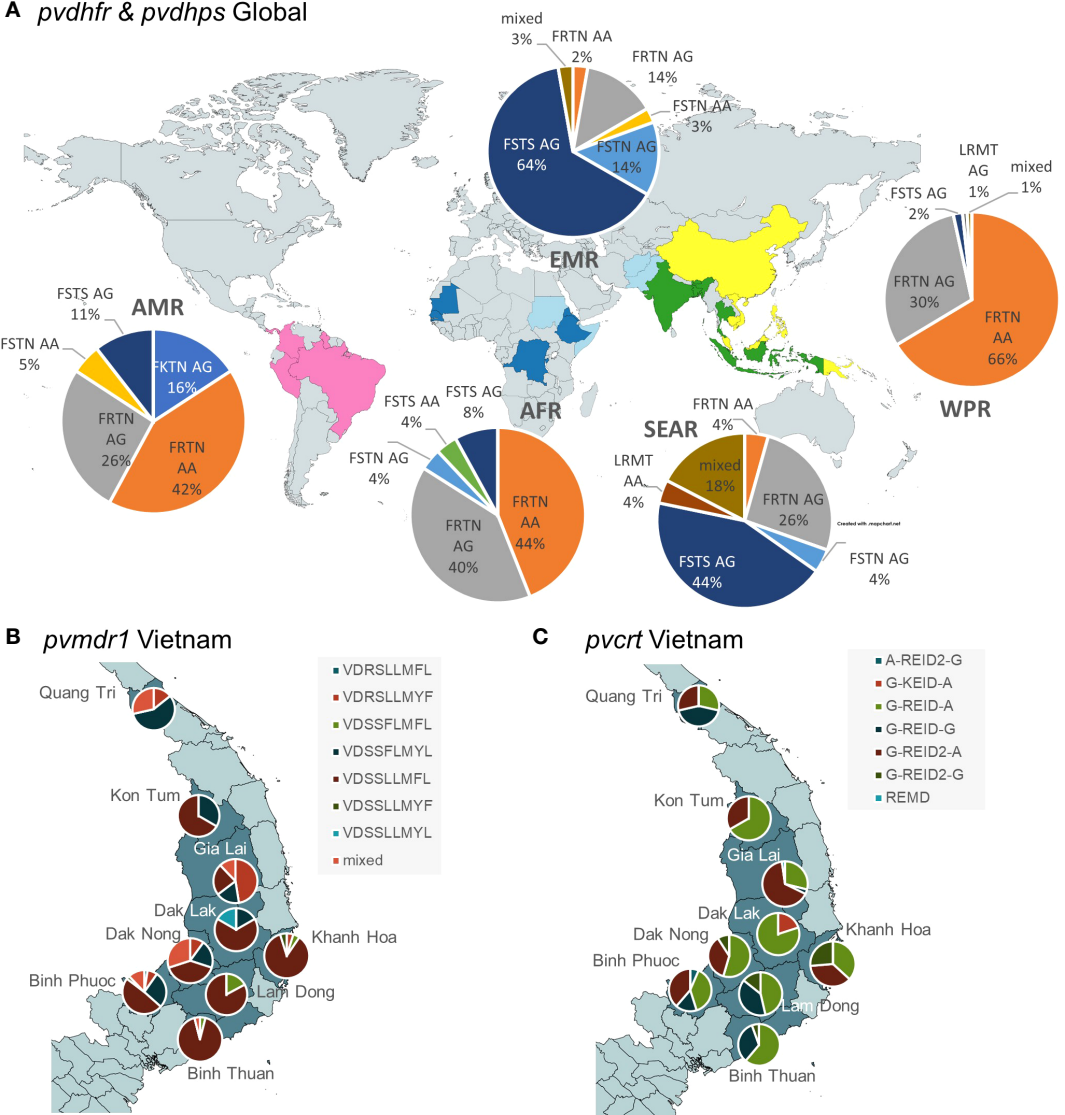
Geographical separation of selected haplotypes in drug resistance associated genes genotyped by Pv AmpliSeq assay. **(A)** Global spread of pvdhps and pvdhfr validated mutations for SP resistance (dhfr: F57L,S58R/K,T61M,N117T/S; dhps: A383G, A553G). Map background created in https://www.mapchart.net/ **(B)** Spread of pvmdr1 haplotypes (V221L, D500N, S513R, S698G, L845F, L908M, T958M, Y976F, F1076L) and **(C)** pvcrt haplotypes (intron 357 + 83G>A, R121K, E207Q, I319M, D328D(A>G), intron 1003-46G>A) at a provincial level in Central Vietnam. Background map created in QGIS v3.16 with spatial data from https://www.diva-gis.org/.

Global widespread sulfadoxine-pyrimethamine (SP) resistance in *P. vivax* has been previously reported (Tjitra et al., 2002; Hastings et al., 2005; Auliff et al., 2006; Rungsihirunrat et al., 2008; Cubides et al., 2018). We observed only one sample from Eritrea with wildtype alleles in *pvdhfr* (FSTS) and *pvdhps* (AA). Single mutants in *pvdhfr* and *pvdhps* (FSTS AG) were predominant in Eastern Mediterranean Region (EMR) and South East Asia Region (SEAR), while double mutants (FRTN AA or FSTN AG) and triple mutants (FRTN AG or FKTN AG) were predominant in the Americas Region (AMR), African Region (AFR) and Western Pacific Region (WPR) (**Figure 6A**). Different nucleotide changes in *pvdhfr* were responsible for the same amino acid mutations in different areas, showing independent emergence of these phenotypes. The amino acid change S58R in *dhfr* haplotypes FRTN and LRMT was caused by a nucleotide change from C>A (type 1) or C>G (type 2). While type 2 is found globally, the type 1 variant was observed only in South America, in co-existence with the type 2 variant at equal frequencies in Peru and Colombia.

### Vietnam population description

**Figure 7:**
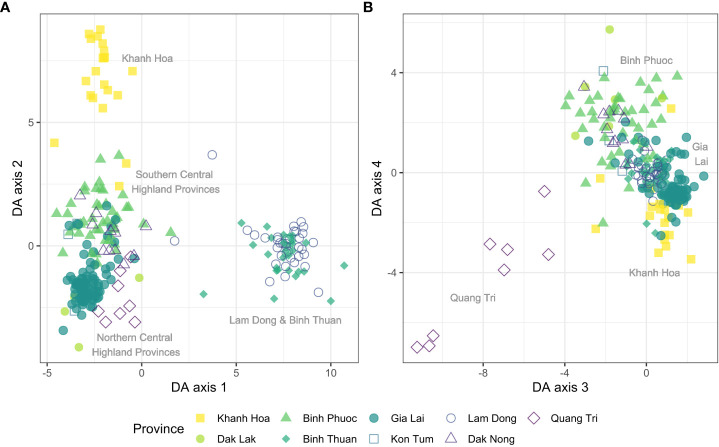
Discriminant analysis of principal components (DAPC) of Vietnam isolates (n = 261) grouped by province. Scatter plot of DA eigenvalues 1 and 2 **(A)** using all biallelic SNPs detected by the Pv AmpliSeq showed a differentiation of Lam Dong and Binh Thuan along the x-axis and a separation of Khanh Hoa province along the y-axis. Scatter plot of DA eigenvalues 3 and 4 **(B)** groups separately clusters isolates from the most Northern province of Quang Tri. SNPs contributing most to the DAPC are listed in **Supplementary Table 2**, **Table S6**. DAPC was performed with 60 principal components and 8 discriminants as determined through cross-validation.

Within Vietnam, we explored the geographic separation at the provincial level, as at that level many of the control interventions are organized. The DAPC was performed on all biallelic SNPs and a clustering of highland provinces *vs.* coastal provinces was seen in the first two DAPC components (**Figure 7**). Among the coastal provinces, parasites in Lam Dong and Binh Thuan were genetically similar, but different from parasites in Khanh Hoa Province. From the variants contributing most to the DAPC across the first four axes, 10/18 are from the 42-SNP Vietnam barcode amplicons, demonstrating the value of the barcode for within-country distribution of isolates (**Supplementary File 2**, **Table S6**). Other alleles contributing to the separation within Vietnam include variants in *pvp13K, pvmdr1, pvmrp2, pvcrt, pvmdr2, pvdhps* and *pvdhfr*. While the Vietnam isolates almost all had the *pvdhfr* haplotype FRTN type 2 (and one isolate with FSTS in Quang Tri), they varied in *pvdhps* haplotypes with high proportions of the G allele in the Northern Provinces.

### Use case drug resistance within Vietnam

Besides *dhfr* and *dhps* there are no clear markers validated for *in vitro* or clinical resistance to CQ or other drugs. However, population studies have uncovered potential CQR markers for both *P. falciparum* and *P. vivax*, and it is therefore important to continue monitoring mutations that could be relevant for resistance.

Here, we observed distinct *pvmdr1* haplotypes at nine amino acid positions in Vietnam, with clear differences between provinces (**Figure 6B**). *Pvcrt* is the orthologue of *P. falciparum* chloroquine resistant marker, but associations with CQR are ambiguous. In Vietnam, similar to other countries, we observed the REID *pvcrt* variants (amino acid positions 121, 207, 319, 328) in most samples. However, there is a frequent silent mutation with a different nucleotide encoding for aspartic acid (D) allele at amino acid position 328, which is prevalent throughout our samples in the WPR and also to a lesser extent in SEAR. Very few other non-synonymous variants in *pvcrt* were observed, while this gene has many intronic variants (Supplementary file 4), two of which contributed to the within country DAPC in Vietnam (**Figure 7**). We observed different haplotypes including these two intronic variants within Vietnam across coastal and highland provinces (**Figure 6C**).

Variants in *pvmrp2* and *pvmdr2* also contributed to the DAPC axes but were not further explored in detail. Genotypes of all variants of interest (all non-synonymous alleles in the 11 drug resistance genes detected in ≥2 samples) can be interactively explored at: https://microreact.org/project/k86kAAWw9Z8PNeUYBj9bvh-plasmodium-vivax-ampliseq-vietnam-and-global.

### Use case transmission intensity

**Figure 8:**
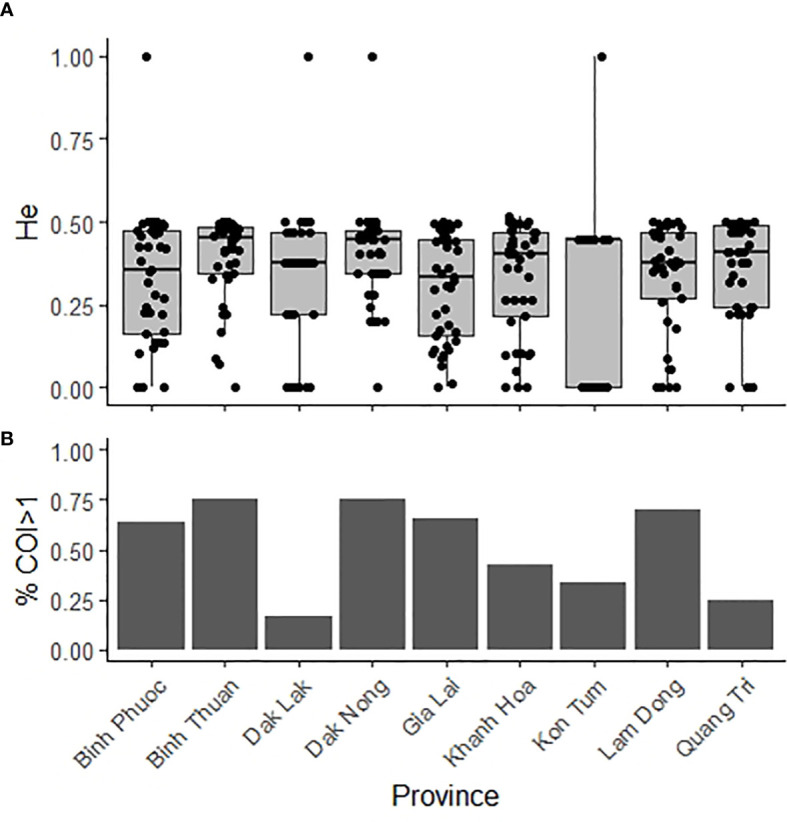
Genetic diversity and complexity of infection in nine provinces in Vietnam 2015-2019. **(A)**. Expected heterozygosity of 42-SNP Vietnam barcode positions **(A)** and proportion of multiple clone infections **(B)** in samples (n = 261) in Vietnam. He = Expected heterozygosity; COI = complexity of infection.

Genetic diversity and the proportion of multiple clone infections are used as measure of transmission intensity (Escalante et al., 2015). We do not observe a large variation in genetic diversity (*He)* at 42-SNP Vietnam barcode positions in the different provinces in Vietnam (**Figure 8**). Samples were collected at selected sentinel sites with similar moderate transmission levels, with provincial level incidence rates varying from 0.11-0.56 per 1000 person-year-at-risk. Median *He* is highest in Binh Phuoc (highest incidence) and Dak Nong (among the lowest incidence). In Gia Lai province, despite a high number of cases, diversity was lower relative to other provinces. For *P. vivax* it is known that other forces besides transmission intensity can contribute to high genetic diversity, such as a high proportion of multiple clone infections that drive recombination, as well as gene flow between different areas. Multiple clone infections were common in most provinces, except in Dak Lak and Quang Tri.

### Use case connectivity of parasite populations

The connectivity between provinces in Vietnam was assessed by measuring to what extent the parasite populations are genetically related. We used pairwise genetic differentiation (F_ST_) between populations as well as pairwise IBD between samples within and between provinces as a measure of connectivity and parasite gene flow in Vietnam.

**Figure 9:**
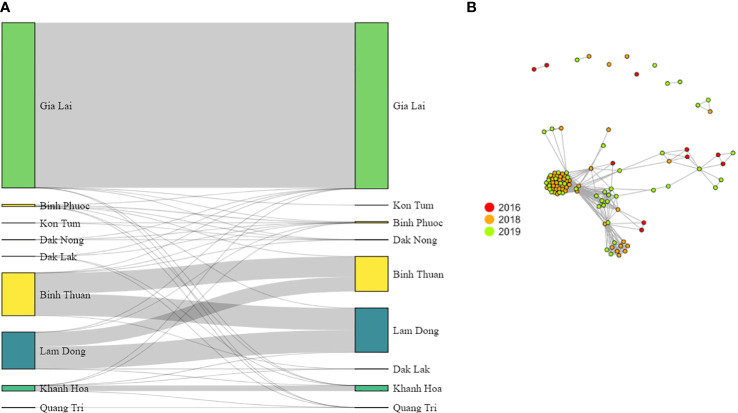
*P. vivax* parasite connectivity in Vietnam. **(A)** Summed pairwise IBD-sharing of isolates between and within provinces. **(B)** Connectivity networks inferred by IBD between *P. vivax* isolates from Gia Lai province. Edges connecting parasite pairs indicate that >60% of their genomes descended from a common ancestor without intervening recombination. Node colors indicate the year of collection.

High connectivity was observed between Lam Dong and Binh Thuan provinces with little genetic differentiation (**Table 5**) and a large pairwise IBD between samples (**Figure 9A**). Lam Dong and Binh Thuan appeared isolated from the other provinces (even with the coastal province of Khanh Hoa) with large genetic differentiation and little IBD sharing. Khanh Hoa had little connectivity with neighboring Dak Lak province, although the samples included were collected in 2015. Conversely, most Central Highland provinces (*i.e.* Dak Lak, Dak Nong, Gia Lai, Kon Tum) and Binh Phuoc showed a small level of connectivity with low level IBD-sharing and lower F_ST_ (**Table 5**). Binh Phuoc (highest incidence) and Dak Nong (among the lowest incidence) showed little differentiation and a small level of IBD-sharing with most other provinces, indicating a more admixed and less isolated parasite population. In Gia Lai province, which contributed the highest number of samples, we observed very little connectivity with other provinces, but very high relatedness (IBD) between samples within the province. This explains the relatively lower diversity in this province despite the high number of cases. The population within Gia Lai province is characterized by multiple clusters of similar isolates, as seen by the IBD-sharing (**Figure 9B**). Some of these clusters of isolates were observed in both 2018 and 2019 years, whereas others only in one year.

**Table 5:** Genetic differentiation among *P. vivax* population in different provinces in Vietnam.

	Kon Tum	Gia Lai	Dak Lak	Dak Nong	Binh Phuoc	Khanh Hoa	Lam Dong	Binh Thuan
Quang Tri	0.1224	0.1791	0.0293	0.0687	0.0344	0.0598	0.1805	0.1963
Kon Tum		0.1623	0.0887	0.0519	0.0390	0.1029	0.1294	0.1709
Gia Lai			0.1473	0.1576	0.0931	0.1413	0.2425	0.2318
Dak Lak				0.0475	0.0253	0.1132	0.2222	0.2498
Dak Nong					0.0230	0.0630	0.1023	0.1419
Binh Phuoc						0.0591	0.1420	0.2100
Khanh Hoa							0.1884	0.1542
Lam Dong								0.0281

Pairwise F_ST_ values (Weir and Cockerham) were estimated with 1,000 bootstraps using the diveRsity package in R.

## Discussion

A new highly-multiplexed sequencing assay for *P. vivax* was successfully designed targeting 11 putative antimalarial drug resistance genes, combined with a 33-SNP vivaxGEN-geo global barcode for prediction of origin and a 42-SNP Vietnam-specific barcode for population genetic analysis. One of the advantages of a targeted approach is that it generates a much higher depth of coverage (100-1000 fold *vs*. 50-100 fold) at a lower cost than WGS, maximizing the use of available sequencing output. The AmpliSeq assay makes it possible to sequence many more samples in one run without leveraging the high depth needed to detect minority clones with phenotypically important characteristics such as drug resistance. The Pv AmpliSeq assay was validated using isolates from travelers and migrants as well as samples from sentinel site surveillance in Vietnam. The assay detected both alleles at the majority (73/75) of barcode loci and achieved good spatial specificity for between-country prediction of origin, and high resolution for within-country diversity and gene flow analysis in Vietnam. Many variants were detected in (putative) drug resistance genes, with spatial distribution of haplotypes, both globally and within Vietnam. The Pv AmpliSeq assay is the first of its kind for molecular surveillance of *P. vivax*. Other NGS assays for *P. vivax* relatedness and population genetic analysis have previously been designed (Lin et al., 2015; Friedrich et al., 2016), but none combine these markers with phenotypic markers in a single assay. Variation in drug resistance associated genes has so far primarily been performed with single target assays or WGS (Auburn et al., 2016; Gresty et al., 2019; Ferreira et al., 2021).

During assay development we prioritized feasibility in- and applicability to researchers and control programs in endemic countries. The Pv AmpliSeq assay achieved good performance on DBS samples down to a parasite density of 5 p/µL, making this type of assay suitable for use in a wide range of settings in malaria endemic countries. DBS samples and microscopy slides collected from (suspected) malaria cases in remote communities are easily transported to a central laboratory, as has been done in this study, and subsequently processed and analyzed. Another potential option could be to store RDTs after diagnosis for subsequent DNA extraction and qPCR and AmpliSeq analysis in a central laboratory (Cnops et al., 2011). The required laboratory infrastructure for the Pv AmpliSeq assay has recently been established at NIMPE in Vietnam and bioinformatic training will be completed in the near future, thus making this valuable tool available to local researchers and the NMCP. With routine sample collections at sentinel sites across Vietnam already ongoing, the next step will be to implement the Pv AmpliSeq assay to generate genetic data on a regular basis (e.g. twice yearly) and standardize reporting to the NMCP to efficiently translate genetic surveillance results into policy-advice for relevant stakeholders.

Raw data resulting from the sequencing can be processed automatically using the software of the sequencer. Subsequent variant analysis can be performed with minimal bioinformatic skills using any software that can analyze tabulated data, however, interpretation of data requires skilled population geneticists. Here, we used a linux-based pipeline that gave us more flexibility and insight during the validation. If detecting variants of minority clones at low proportions is important for the downstream analysis, additional variant calling approaches (e.g. Dada2, HaplotypR, etc.) can be explored, although these require advanced bioinformatic skills and validation. An automated analysis environment would be of great benefit for rapid output of genetic reports for NMCPs and is currently under development. For the prediction of origin, the genotyped barcodes can be used as input for a previously described online predictor tool, vivaxGEN-geo (https://geo.vivaxgen.org/) (Trimarsanto et al., 2019). The output from vivaxGEN-geo is a simple prediction of the most likely country of origin that can be easily interpreted by individuals who may have limited knowledge of genetic epidemiology (*i.e.*, without the need for interpreting complex plots such as phylogenetic trees or PCoAs), enabling them to make decisions on appropriate case management responses.

The Pv AmpliSeq assay included two different barcodes with different purposes, the vivaxGEN-geo with specific alleles for between-country analysis and the 42-SNP Vietnam barcode with alleles that were variable between different provinces with high resolution in Vietnam, supported by their contributions in the DAPC.

These barcodes were combined and the previously reported likelihood classifier (Trimarsanto et al., 2019) was used to predict the origin of a unique sample collection originating from many countries across the globe from travelers and migrants. The prediction of origins was accurate at the country level for many countries in South East Asia and the Western Pacific. Predictions were accurate at the regional level in South America, Africa and Central Asia due to existing gaps in the reference genomic dataset used for the predictions. Several travelers’ isolates with a travel history to non-malaria endemic countries were predicted to originate from Central Asia, specifically Afghanistan. As this is the region with highest numbers of *P. vivax* cases (World Health Organization, 2021), this region could contribute highly to imported infections as has been reported in Greece and China (Spanakos et al., 2018; Zhang et al., 2018; Cui et al., 2021) and enhanced by the long latency time of *P. vivax* isolates from this region (Battle et al., 2014). Better predictions can likely be achieved in these areas with more recent genomes with a wider geographic spread in the reference dataset. *P. vivax* malaria in returning travelers has been reported to be relatively common (Elliott et al., 2006), and the current study along with another recent genomics study (Benavente et al., 2021), show that travel clinics and diagnostic reference centers in Europe or other non-endemic countries can serve as source of parasite strains from remote or inaccessible places that would otherwise not be investigated. These isolates can be an important resource to fill in the gaps in genetic and/or genomic databases.

Our approach, by including a country-specific barcode, was successful with a high resolution for in-country analysis of parasite diversity, population genetics and gene flow, in contrast to other previously reported more general barcodes (Baniecki et al., 2015; Trimarsanto et al., 2019). The prediction of origin for isolates from Vietnam and Cambodia improved upon the addition of the 40-SNPs from the Vietnam barcode. This barcode will be added as an option to the online predictor tool for predictions in Vietnam and the Greater Mekong Subregion (GMS). However, while 266/269 (98.8%) Vietnamese samples had correct predictions to the broader Vietnam/Cambodia region, many Vietnam samples were predicted to originate from Cambodia and vice versa. This could be due to the lack of population structure and high amount of gene flow between these two countries (Benavente et al., 2021), as a result of frequent human population movements in this region of the GMS (Edwards et al., 2015; Gryseels et al., 2015). At a smaller geographic scale similar high level of connectivity was seen in Lam Dong and Binh Thuan provinces, which share 200km of forest along the provincial borders, where malaria is predominantly caused by *P. vivax*. Human population movement across this border is frequent because of harvesting activities in the forest fields (main source of malaria infections). In addition, both of these provinces are well-known for their culture and local tourism, which could have contributed to the spread and connection between these areas. This has important consequences for the Vietnamese and regional strategy for *P. vivax* elimination and indicates the limited use of prediction of origin tools while cross-border migration of parasites is frequent. While we set out to design a country specific barcode, allele frequencies and similarities between populations observed here indicate that the current 42-SNP barcode might have sufficient resolution in neighboring countries in the GMS, although this requires further validation in future studies.

In contrast, a high rate of shared ancestry was observed in Gia Lai province, which is a relatively remote province with the highest malaria incidence (both *P. falciparum* and *P. vivax)* in Vietnam. Krong Pa district (where most of the samples were collected) has an ethnic minority population living and working in the near vicinity of the health center (six communes that are at most 20km apart) who do not frequently travel out of the area. As Vietnam and neighboring countries progress further towards elimination, malaria will likely become more focal, and prediction tools will be of a greater benefit to malaria control, as for example in China where all cases are now imported (Zhang et al., 2018; Feng et al., 2020; Cui et al., 2021).

For other countries or regions, the same SNP barcode design approach used here can be applied. SNPs of interest can be submitted for rapid amplicon design and combined with existing amplicons in the Pv AmpliSeq assay. This way the new primers are combined in new primer pools, and the benefit of the AmpliSeq technology is that it does not require individual amplicon optimization. In parallel we are in the process of validating another version of the Pv AmpliSeq, which has a different within-country barcode designed with genomes from Peru (manuscript in preparation) with potential applicability to the wider Amazon basin. For other countries where it is not feasible to design a specific within-country barcode, allele frequencies of existing Pv AmpliSeq barcodes can be evaluated using the resources provided in this manuscript (supplementary file S5) or other genomic resources such as the MalariGEN datasets (Adam et al., 2022), if the country or region is represented in these datasets. Ideally, MAF should vary between 0.1-0.5 with differences in MAF at smaller geographic scales within the country or region.

The Pv AmpliSeq detected many variants in the 11 putative drug resistance genes, and the data generated in this study (variants of interest list in Supplementary file 4) with its wide geographic range, can serve as a data resource for global variation in these genes. Non-synonymous variants in genes contributing to global spread, as well as within Vietnam, (variants in *pvmdr1*, *pvmdr2*, *pvdmt2*, *pvmrp2*, *pvcrt*, and *pvp13K*) can be prioritized in studies aiming to characterize drug resistant phenotypes, for example in model systems such as transgenic *P. knowlesi*. Mutations in *pvmdr1* have recently been shown in such a model to be associated with reduced sensitivity to mefloquine, dihydroartemisinin, and lumefantrine (L. Buyon et al. 8th International Conference on *Plasmodium vivax* Research April 2022). Selection of different *pvmdr1* variants within Vietnam could be the result of treatment of co-endemic *P. falciparum*, which has also undergone strong ART selection, especially in the central highland provinces [(Hamilton et al., 2019) and unpublished data Rovira-Vallbona et al.]. Since the design of the Pv AmpliSeq assay, several studies have implied the association of *pvcrt* expression and chloroquine resistance (CQR) and associated it with markers in the promotor region (Sá et al., 2019). The promotor region of *pvcrt* together with *pvkelch10* (found to be under positive selection in a recent genomics study) (Benavente et al., 2021) could be targeted in a new version of the Pv AmpliSeq assay as the design can be easily adapted.

While the assay is more cost-efficient than WGS [0.6x the cost (Kattenberg et al., 2021)], the price can still surpass the budgets available at NMCPs for genetic surveillance. It can be further reduced by pooling up to three samples into one library preparation reaction, resulting in comparable population level allele frequencies, and reducing the cost 3-fold (making it 0.2x the cost of WGS). Sample pooling might be a cost-effective strategy when determining the prevalence of drug resistance associated mutations. Other options that could be explored in order to reduce costs include: increasing the number of samples that are sequenced in one run from 144 to 384 (this will reduce the depth of coverage per sample, although it will be more than enough (~100X) for most applications); explore the performance of the assay with reagents from other supplies and/or different sequencing platforms, such as nanopore sequencing.

In conclusion, Pv AmpliSeq performs well for multiple use cases drawing conclusions on parasite diversity, distribution of parasite resistance and dynamics of parasite populations. In addition, this assay can be easily tailored to fit different new markers and in-country specific barcodes to inform decision making at the country level where NMCP can action genetic epidemiology data to improve and better target interventions at the desired spatial scale and within the local context.

## Data availability statement

The datasets presented in this study can be found in online repositories. The names of the repository/repositories and accession number(s) can be found in the article/**Supplementary Material**.

## Ethics statement

The studies involving human participants were reviewed and approved by Institutional Review Board of the Institute of Tropical Medicine Antwerp. The patients/participants provided their written informed consent to participate in this study.

## Author contributions

Conception of ideas for the study, AR-U, JK, NB, NVH. Selection of targets and design of the assay, AC-P, ES, and JK. Sample collections and coordination of field work, NLH and NVH. Contribution of data and/or resources, ME, NX, and SA. Laboratory experiments, ES, JK, PG, and NN. Bioinformatics and data analysis, JK, HT, and PM. Writing first draft manuscript, ES, JK, and NVH. Supervision, AR-U. All authors reviewed and contributed to the final version of the manuscript.

## Funding

This work was funded by the Belgium Development Cooperation (DGD) under the Framework Agreement Program between DGD and ITM (FA4 Vietnam, 2017-2021). ES is supported by a PhD-fellowship from the Research Foundation - Flanders (FWO). Funding agencies had no role in the design of the study. The research was also funded in part by the Australian National Health and Medical Research Council (APP2001083).

## Acknowledgments

We wish to thank all participants in the study for making their samples available for this research. In addition, we would like to thank all clinical, microscopy, laboratory, field and administrative staff who supported the sample collections and/or this study in any way. The computational resources used for the WGS analyses were provided by the HPC core facility CalcUA of the University of Antwerp, and VSC (Flemish Supercomputer Center). We would also like to thank Dr Ric Price, Dr Awab Ghulam Rahim, Dr Mohammad Shafiul Alam, Dr Wasif A Khan, Dr Sonam Wangchuck, Dr Zuleima Pava, Dr Diego F Echeverry, Mr Sisay Getachew, Dr Abraham Assefa, Dr Beyene Petros, Dr Yaghoob Hamedi, Dr Ishag Adam and Dr Francois Nosten for the generous contribution of DNA samples within the vivaxGEN framework to support the geographic evaluation of the barcode.

## Conflict of interest

The authors declare that the research was conducted in the absence of any commercial or financial relationships that could be construed as a potential conflict of interest.

## Publisher’s note

All claims expressed in this article are solely those of the authors and do not necessarily represent those of their affiliated organizations, or those of the publisher, the editors and the reviewers. Any product that may be evaluated in this article, or claim that may be made by its manufacturer, is not guaranteed or endorsed by the publisher.
