# Supplementary material for: Novel highly-multiplexed AmpliSeq targeted assay for Plasmodium vivax genetic surveillance use cases at multiple geographical scales
Source: Front Cell Infect Microbiol. 2022 Aug 11;12:953187. doi: 10.3389/fcimb.2022.953187 (PMC9403277; doi:10.3389/fcimb.2022.953187)

**Supplementary File 2 with supplementary figures and tables**

**Supplementary table S1.** Sentinel site DBS collection overview inclusion in AmpliSeq

|  | **2018** | | | **2019** | | |
| --- | --- | --- | --- | --- | --- | --- |
|  | **Excl.** | **Incl.** | **% incl.** | **Excl.** | **Incl.** | **% incl.** |
| Binh Phuoc | 25 | 29 | 54% | 8 | 12 | 60% |
| Binh Thuan | 2 | 6 | 75% | 19 | 27 | 59% |
| Dak Nong |  |  |  | 0 | 15 | 100% |
| Gia Lai | 41 | 49 | 54% | 49 | 49 | 50% |
| Khanh Hoa | 2 | 7 | 78% | 8 | 14 | 64% |
| Kon Tum | 2 | 3 | 60% |  |  |  |
| Lam Dong | 9 | 16 | 64% | 11 | 23 | 68% |
| Ninh Thuan | 0 | 1 | 100% |  |  |  |
| Quang Tri |  |  |  | 5 | 8 | 62% |

**Supplementary table S2**. *P. vivax* 18S qPCR primers.

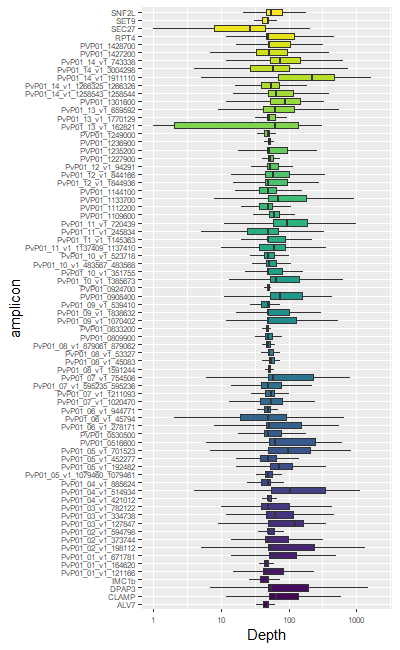


**Supplementary figure S1.** Mean depth of coverage after filtering for each barcode amplicon.


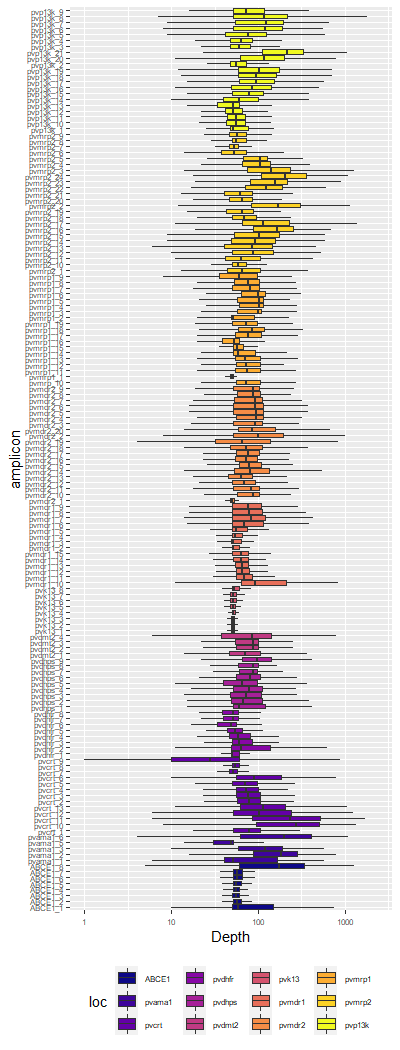


**Supplementary figure S2.** Mean depth of coverage after filtering for each drug resistance and *ama1* amplicon. Color scales are for the locus or gene targeted by the amplicon.

**Supplementary table S3.** Sample dilutions to determine the density limit of the assay. The same dilutions were tested with and without prior sWGA.

|  | **parasite density (p/µL)** | **aligned coverage** | | | **% genotypes missing** | | |
| --- | --- | --- | --- | --- | --- | --- | --- |
|  |  | normal | sWGA | sWGA diluted input | normal | sWGA | sWGA diluted input |
| PV LM quantified | 20000 | 263.6 | 7.5 |  | 1.4% | 68.7% |  |
|  | 2000 | 1486.6 | 14.4 | 589.4 | 0.4% | 52.1% | 70.8% |
|  | 200 | 1175.1 | 80.7 | 806.4 | 0.3% | 22.2% | 5.4% |
|  | 20 | 112.2 | 904.8 | 175.1 | 3.7% | 63.8% | 3.8% |
|  | 2 | 4.7 | 395.3 |  | 93.6% | 83.5% |  |
|  | 0.2 | 8.4 | 268.2 |  | 98.6% | 91.6% |  |
| Low density Pv from DBS Vietnam | 51.6 | 1564.4 | 100.7 |  | 1.9% | 10.8% |  |
|  | 5.2 | 323.9 | 130.4 |  | 1.9% | 30.1% |  |
|  | 0.5 | 33.8 | 749.2 |  | 54.9% | 82.3% |  |
|  | 0.05 | 8.6 | 999.2 |  | 95.5% | 93.9% |  |
|  | 0.005 | 8.8 | 279.7 |  | 99.4% | 85.6% |  |


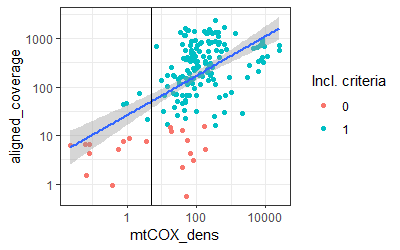


**Supplementary figure S3.** Parasite density by mtCox1 qPCR (in parasites/µL) vs. coverage achieved in the Pv AmpliSeq assay, with linear regression line in blue. Samples colored in red did not pass the inclusion criteria (coverage >15 and missing genotypes <50%). The horizontal black line denotes the limit of 5 p/µL, below which the majority of samples do not pass the inclusion criteria. Samples that failed above this threshold were either misquantified or failed due to another reason.

**Supplementary table S4.** Consensus Sanger sequences compared to Pv AmpliSeq genotypes at 3 *pvmdr1* loci

| **Sanger** | **AmpliSeq** | | |  |  |
| --- | --- | --- | --- | --- | --- |
|  | matched genotype | mixed | incorrect | missing | % correct |
| *pvmdr1* F1076L | 114 | 8 | 0 | 0 | 93.4% |
| *pvmdr1* Y967F | 115 | 0 | 0 | 8 | 100.0% |
| *pvmdr1* T958M | 120 | 0 | 0 | 2 | 100.0% |
|  |  |  |  | overall | 97.8% |

**Supplementary table S5. Contributions of loci to the DAPC first four DA eigenvalues of the global dataset.**

| **Target** | **variant** | **position** | **axis** | **contributing value** |
| --- | --- | --- | --- | --- |
| pvmdr2 | Val43Leu | PvP01_12_v1_2441608 | 1 | 0.0154 |
| **vivaxGEN-geo barcode** |  | PvP01_11_v1_1448769 | 1 | 0.0138 |
| VTN barcode amplicon |  | PvP01_05_v1_701489 | 1 | 0.0126 |
| pvdmt2 | Ile28Ile (syn) | PvP01_03_v1_552932 | 1 | 0.0119 |
| **vivaxGEN-geo barcode** |  | PvP01_14_v1_1266326 | 1 | 0.0107 |
| pvmrp1 | Leu1207Ile | PvP01_02_v1_155305 | 1 | 0.0106 |
| **vivaxGEN-geo barcode amplicon** |  | PvP01_12_v1_1116522 | 1 | 0.0103 |
| pvmdr2 | Val468Val (syn) | PvP01_12_v1_2442885 | 1 | 0.0100 |
| **vivaxGEN-geo barcode** |  | PvP01_08_v1_442363 | 2 | 0.0224 |
| pvmdr1 | Leu1076Phe | PvP01_10_v1_479908 | 2 | 0.0176 |
| **vivaxGEN-geo barcode** |  | PvP01_06_v1_646186 | 2 | 0.0156 |
| **vivaxGEN-geo barcode amplicon** |  | PvP01_11_v1_1448967 | 2 | 0.0145 |
| pvmdr2 | Val851Val | PvP01_12_v1_2444034 | 2 | 0.0144 |
| pvmrp2 | Tyr62Cys | PvP01_14_v1_2057938 | 2 | 0.0141 |
| **vivaxGEN-geo barcode** |  | PvP01_12_v1_1400307 | 2 | 0.0135 |
| **vivaxGEN-geo barcode amplicon** |  | PvP01_06_v1_646355 | 2 | 0.0121 |
| pvdhps | Met205Ile | PvP01_14_v1_1271444 | 3 | 0.0323 |
| **vivaxGEN-geo barcode amplicon** |  | PvP01_06_v1_646176 | 3 | 0.0286 |
| VTN barcode amplicon |  | PvP01_12_v1_1844886 | 3 | 0.0161 |
| **vivaxGEN-geo barcode** |  | PvP01_06_v1_646186 | 3 | 0.0160 |
| pvdhfr | Arg58Ser | PvP01_05_v1_1077535 | 3 | 0.0144 |
| pvdhfr | Asn117Thr | PvP01_05_v1_1077711 | 3 | 0.0140 |
| VTN barcode |  | PvP01_06_v1_278171 | 4 | 0.0161 |
| VTN barcode amplicon |  | PvP01_13_v1_659611 | 4 | 0.0160 |
| **vivaxGEN-geo barcode** |  | PvP01_14_v1_1266326 | 4 | 0.0135 |
| VTN barcode amplicon |  | PvP01_02_v1_198202 | 4 | 0.0135 |
| VTN barcode |  | PvP01_11_v1_1145363 | 4 | 0.0123 |
| **vivaxGEN-geo barcode** |  | PvP01_14_v1_344881 | 4 | 0.0121 |
| pvmrp1 | Asn568Asn (syn) | PvP01_02_v1_157220 | 4 | 0.0103 |

**Supplementary table S6. Contributions of loci to the DAPC first four DA eigenvalues of the Vietnam dataset.**

| **Target** | **variant** | **position** | **axis** | **contributing value** |
| --- | --- | --- | --- | --- |
| pvp13K | Tyr676His | PvP01_10_v1_829461 | 1 | 0.0240 |
| **VTN barcode amplicon** |  | PvP01_12_v1_844299 | 1 | 0.0180 |
| **VTN barcode amplicon** |  | PvP01_11_v1_1145363 | 1 | 0.0154 |
| pvmdr1 | Phe976Tyr | PvP01_10_v1_480207 | 1 | 0.0119 |
| pvmrp2 | Ala1869Glu | PvP01_14_v1_2052517 | 1 | 0.0103 |
| **VTN barcode amplicon** |  | PvP01_03_v1_127960 | 1 | 0.0101 |
| **VTN barcode** |  | PvP01_03_v1_334738 | 2 | 0.0234 |
| **VTN barcode** |  | PvP01_14_v1_1911110 | 2 | 0.0154 |
| pvmdr1 | Phe976Tyr | PvP01_10_v1_480207 | 2 | 0.0143 |
| **VTN barcode amplicon** |  | PvP01_06_v1_45965 | 2 | 0.0139 |
| pvcrt | intron variant | PvP01_01_v1_444779 | 2 | 0.0119 |
| **VTN barcode** |  | PvP01_03_v1_782122 | 3 | 0.0245 |
| **VTN barcode amplicon** |  | PvP01_07_v1_1211080 | 3 | 0.0155 |
| pvmdr2 | Thr1467Met | PvP01_12_v1_2445881 | 3 | 0.0100 |
| **VTN barcode** |  | PvP01_10_v1_1385736 | 4 | 0.0164 |
| **VTN barcode** |  | PvP01_04_v1_885624 | 4 | 0.0144 |
| pvdhps | G383A | PvP01_14_v1_1270911 | 4 | 0.0135 |
| pvdhps | intron variant | PvP01_14_v1_1272164 | 4 | 0.0112 |
| vivaxGEN-geo barcode amplicon |  | PvP01_05_v1_1285244 | 4 | 0.0106 |
| pvdhps | intron variant | PvP01_14_v1_1269978 | 4 | 0.0100 |

**Supplementary figure S4. Comparison of country prediction performance between the 33-SNP and 72-SNP panels.** The boxplots present Matthews Correlation Coefficient (MCC) scores as a measure of prediction accuracy, which can range from -1 (total disagreement) to 1 (perfect prediction). MCC scores were generated from the likelihood classifier using 500 repeats with stratified 10-fold cross validation for each SNP set. MCC scores plotted for only the vivaxGEN-geo barcode (GEO-33, dark-blue) *vs.* the vivaxGEN-geo barcode with the 40-SNPs from the VTN barcode (ANTWERP-73, light blue). The combined barcodes perform better for many countries, especially in South East Asian and Western Pacific countries, incl. Vietnam, Cambodia, Thailand, Myanmar and Indonesia. However, the combined barcodes perform slightly worse for South American countries, especially Brazil.


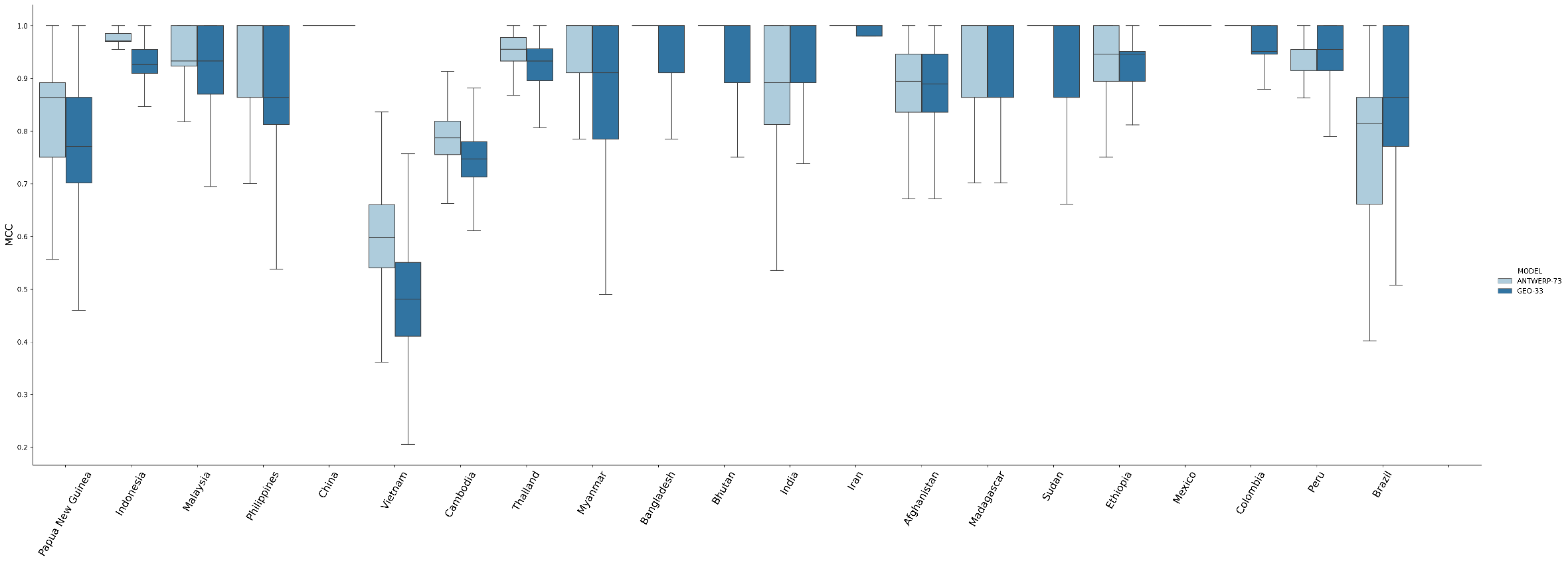

Supplement: Supplementary file 2 [file Table_2.docx]
